# Label-Free Proteomics Reveals the Response of Oat (*Avena sativa* L.) Seedling Root Respiratory Metabolism to Salt Stress

**DOI:** 10.3390/ijms26062630

**Published:** 2025-03-14

**Authors:** Xiaojing Chen, Baoping Zhao, Junzhen Mi, Zhongshan Xu, Jinghui Liu

**Affiliations:** 1 National Outstanding Talents in Agricultural Research and Their Innovative Teams, Hohhot 010019, China; 13214088889@163.com (X.C.); zhaobaoping82@163.com (B.Z.); mijunling1206@126.com (J.M.); 2 Cereal Engineering Technology Research Center, Inner Mongolia Autonomous Region, Hohhot 010019, China; 3 College of Life Sciences, Inner Mongolia Agricultural University, Hohhot 010019, China

**Keywords:** oat, salt stress, label-free, proteomics, respiratory metabolism

## Abstract

Soil salinity is among the crucial factors influencing agricultural productivity of crops, including oat. The respiratory metabolic pathways are of great significance for plants to adapt to salt stress, but current research is limited and there are few reports on salt-tolerant crops such as oat, which is necessary to conduct in-depth research. In this study, we conducted a pot experiment to determine the effects of salt stress on oat root growth and respiratory metabolism. Three salt stress levels—control (CK), moderate, and severe—were applied to compare the salt tolerance of the salt-tolerant cultivar Bai2 and the salt-sensitive cultivar Bai5. We selected oat roots at the seedling stage as the research focus and analyzed fresh root samples using an Oxytherm liquid-phase oxygen electrode, a digital scanner, and proteomics. The results showed that with an increased concentration of salt stress, the dry and fresh weight, root–shoot ratio, total root length, root surface area, root volume, and average diameter of the two oat cultivars showed a decreasing trend. Compared with CK, the total root respiration rate of Bai2 under moderate and severe stress decreased by 15.6% and 28%, respectively, and that of Bai5 decreased by 70.4% and 79.0%, respectively. After quantitative analysis of 18 oat root samples from the 2 cultivars using the label-free method, 7174 differential proteins were identified and 63 differential proteins were obtained, which involved 7 functional categories. In total, 111 differential proteins were specifically expressed in the root of the salt-tolerant cultivar Bai2, involving 12 functional categories. Through interaction network analysis, the proteins differentially expressed between the salt treatment and CK groups of the salt-tolerant cultivar Bai2 were analyzed. In total, five types of differentially expressed proteins interacting with each other were detected; these mainly involved antioxidant enzymes, pyruvate metabolism, glycolysis, tricarboxylic acid cycle, and energy metabolism pathways. Salt stress promoted the respiration rate of oat root glycolysis. The respiration rate of the tricarboxylic acid pathway decreased with increased salt stress concentration, while the respiration rate of the pentose phosphate pathway increased. Compared with CK, following moderate and severe salt stress treatment, alcohol dehydrogenase activity in Bai2 increased by 384% and 145%, respectively, while that of Bai5 increased by 434% and 157%, respectively. At increased salt stress concentrations, Bai2 mainly used pyruvate–ethanol fermentation for anaerobic respiration, while Bai5 mainly used pyruvate–lactic acid fermentation for anaerobic respiration. This significant discovery revealed for the first time from the perspective of respiratory metabolism that different salt-tolerant oat cultivars adapt to salt stress in different ways to maintain normal growth and development. The experimental results provide new insights into plant adaptation to salt stress from the perspective of respiratory metabolism.

## 1. Introduction

Abiotic stress includes salt stress, temperature stress, and drought stress [1]. Saline soil represents a major concern, with about 95 million hectares of soil globally, or about 20% of agricultural land, affected by salinity [2]. The total area of salinized land in China is about 36.658 million hectares, and the salinized area of cultivated land reaches 9.209 million hectares, with NaCl and Na_2_SO_4_ as the main components [3]. It is now understood that salt stress inhibits plant growth and development [4], eventually leading to yield reduction and limiting the sustainable development of agricultural productivity in salinized areas. As a dual-purpose grain and feed crop, oat demonstrates tolerance to saline–alkali and barren conditions and drought resistance. It is mainly planted in semi-arid agricultural and pastoral areas in northwest China and high-altitude mountainous areas in southwest China. Importantly, oat has emerged as a leading crop for improving saline–alkali soil [5].

In recent years, a large number of studies have analyzed the effects of salt on plants at the physiological and molecular levels. Current evidence suggests that soil salt causes plants to suffer from osmotic stress, ion toxicity, oxidation stress [6], and other secondary reactions, resulting in various metabolic pathway disorders, including signal transduction, energy metabolism, and hormone synthesis [7]. Roots are the primary organs through which crops absorb water and nutrients, and they are also the first point of contact with salt stress [8]. Their growth and physiological metabolism directly affect the plant’s overall development, yield, and quality. When exposed to salt stress, plant roots adjust their morphology and biomass, trigger physiological and biochemical reactions, and initiate signal transduction pathways to adapt to the environment [9].

As the basis of all life activities, respiratory metabolism provides plants with energy and raw materials for biosynthesis. It involves four key pathways: the Embden–Meyerhof–Parnas (EMP) pathway, the tricarboxylic acid (TCA) cycle pathway, the pentose phosphate pathway (PPP), and the cytochrome pathway (CCP) [10,11]. During seed germination, the EMP and PPP are dominant, with the PPP playing a leading role when mitochondria are active. Conversely, when mitochondrial ATP synthesis is blocked due to hypoxia, the substrate enters the EMP pathway for catabolism [12]. Increasing the PPP-to-EMP-TCA ratio can improve the active oxygen scavenging ability of papaya [13], while studies suggest that the EMP-TCA pathway can promote the TCA cycle and produce more ATP for stress resistance [14]. To prevent cell damage under hypoxic conditions, plant cells initiate various stress responses, including major metabolic rearrangements, hormonal regulation, changes in mitochondrial biology, and gene expression reprogramming [15,16]. These responses involve differences in genes, proteins, transcripts, etc. Through glycolysis, plant cells can be influenced by alcohol dehydrogenase (ADH) and pyruvate decarboxylase (PDC) to simultaneously downregulate genes related to ATP consumption pathways, such as lipid metabolism, secondary metabolism, transport, signaling, and redox regulation [17,18]. It has been established that salinization makes the soil structure compact, reducing permeability and leading to decreased plant root oxygen concentration and the disruption of EMP metabolism, ultimately affecting plant respiration and metabolism [19]. Studies have shown that salt stress can increase the plant respiration rate, significantly affect the TCA cycle pathway of cucumber seedlings, and inhibit the activities of key enzymes in this pathway [20,21,22]. Overexpression of PDC and ADH in Arabidopsis thaliana can improve its tolerance to oxygen deficiency [23], while ADH and PDC function-deficient mutants of maize, rice, and Arabidopsis thaliana are more sensitive to low-oxygen conditions, highlighting the importance of ethanol fermentation in tolerating low-oxygen stress [24,25,26,27]. Although studies have investigated the effects of salt stress on respiratory metabolism, differences exist among different crops. For example, sorghum produced a burst of respiration associated with renewed synthesis of biomass from stored photosynthate under salt stress [28], *S. alterniflora* adapts to constant salinity through fixed, salinity-dependent structural modifications, such as stomatal density [29], while salt-tolerant wheat cultivar exhibited higher respiration rates to resist salt stress [30], therefore necessitating further exploration of changes in respiratory metabolic pathways and key genes. To further investigate the response of oat root respiration metabolism to salt stress, the present study employed a pot experiment to establish three different salt stress concentrations. Two oat genotypes were selected: the salt-tolerant cultivar Bai2 and the salt-sensitive cultivar Bai5. Seedling roots were subjected to various salt stress levels. Root growth, respiratory rate, pathway contribution rates (EMP, TCA, and PPP), and the activity of related enzymes were measured. Through proteomic analysis, the main metabolic pathways and key proteins were investigated to elucidate the mechanism of adaptation of oat to salt stress from the perspective of respiratory metabolism. This research aims to provide a theoretical basis and technical guidance for breeding salt-tolerant oat cultivars and promoting green, high-yield cultivation.

## 2. Results

### 2.1. Growth of Oat Seedlings Under Salt Stress

**Figure 1:**
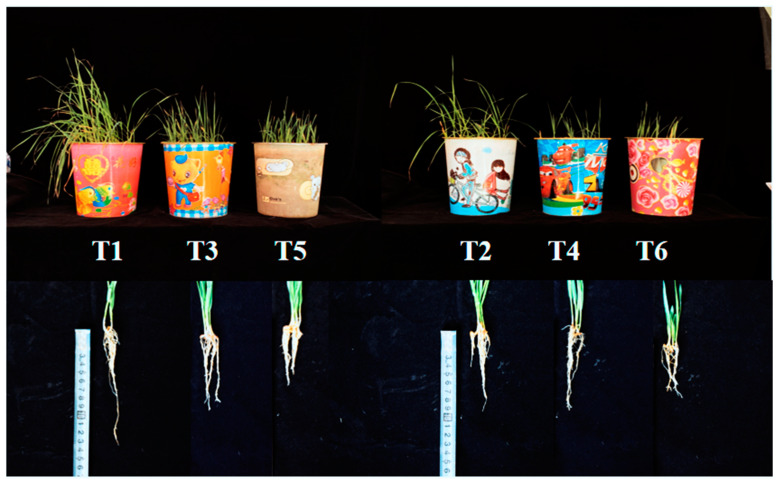
Phenotype of oat under salt stress. Note: T1, T3, T5 and T2, T4, T6 represent CK (0 mM), moderate stress (100 mM), and severe stress (150 mM) of Bai2 and Bai2, respectively. In the following figures and tables, they represent the same meanings.

Figure 1 showed the differences in growth between two oats with different salt tolerance after salt stress. Moderate and severe salt stress had a negative impact on the growth of both oat cultivars, and the more severe the stress, the greater the impact. In addition, the harm caused by salt stress to the growth of salt-tolerant cultivars is relatively small. As the salt stress concentration increased, a gradual decrease in the fresh weight of both oat cultivars was observed. Compared to T1, the fresh weight of T3 and T5 plants declined by 17.8% and 24.4% in the aboveground parts, respectively, and by 45.5% and 54.5% in the underground parts, respectively. Similarly, compared to T2, the fresh weight of T4 and T6 plants showed reductions of 13.2% and 32.1% in the aboveground parts, respectively, and 64.3% and 71.4% in the underground parts, respectively (Table 1). The dry weight of both aboveground and underground parts followed the same decreasing trend as the fresh weight. Interestingly, compared to T1, T3 exhibited a 2.1% increase in aboveground dry weight, while T5 displayed a 6.4% decrease after T5. Both T3 and T5 exhibited reductions in the root cap ratio compared to T1, with decreases of 55.6% and 33.3%, respectively. Similarly, T4 and T6 displayed reductions of 57.7% and 57.7%, respectively, compared to T2. Notably, under moderate and severe stress conditions, the reductions in dry and fresh weight of both aboveground and underground parts, as well as the root–shoot ratio, were less pronounced in Bai2 compared to Bai5 (Table 2).

The roots were crucial for crops to absorb nutrients, grow and develop, and achieve high yields, while root growth in oat seedlings was inhibited by increasing salt stress concentrations. The total root length, root surface area, root volume, and average root diameter were significantly affected by different salt stress concentrations (*p* < 0.05). Compared to T1, T3 and T5 demonstrated reductions of 17.6% and 36.6% in root length, respectively, while T4 and T6 demonstrated reductions of 22.5% and 40.6%, respectively, compared to T2. Similar trends were observed for root surface area, with T3 and T5 showing reductions of 23.8% and 42.8% and T4 and T6 showing reductions of 27.1% and 44.6% compared to their respective controls. Root volume reductions were even more pronounced, with T3 and T5 plants exhibiting reductions of 27.9% and 61.9% and T4 and T6 plants exhibiting reductions of 39.4% and 74.8% compared to their respective controls. The effect on average root diameter was less pronounced, with T3 and T5 showing reductions of 3.2% and 9.7% and T4 and T6 showing reductions of 6.6% and 11.5% compared to their respective controls. Notably, the decrease in root morphological indexes was greater under severe salt stress (T5 and T6) compared to moderate stress (T3 and T4). Additionally, for each stress concentration, Bai2 had higher values for all root morphological indices compared to Bai5 (Table 3).

### 2.2. Effects of Salt Stress on Potassium, Sodium, Calcium, and Magnesium Ion Contents in Oat Root at Seedling Stage

**Figure 2:**
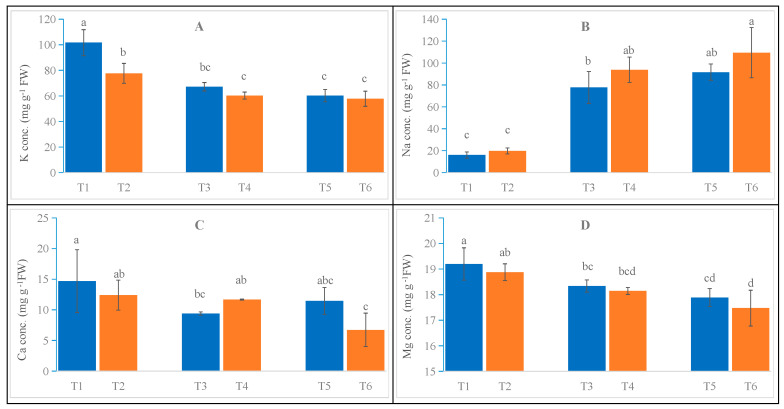
Physiological changes in the roots of Bai2 and Bai5 under normal and salt stress conditions. Contents of K^+^ (**A**), Na^+^ (**B**), Ca^2+^ (**C**), and Mg^2+^ (**D**) were determined in the roots of Bai2 and Bai5 after salt treatments and CK. Data are the mean ± SD values of three biological replicates (*n* = 3), and different letters indicate significant difference at *p* < 0.05, determined via one-way ANOVA.

Similar to the results of salt stress on oat root morphology mentioned above, in both oat cultivars, the potassium ion content exhibited a decreasing trend with increasing salt stress concentration, as illustrated in Figure 2A. Compared to T1, T3 and T5 showed reductions of 33.9% and 40.8%, respectively, while the potassium ion content in T4 and T6 was 22.4% and 25.6% lower, respectively, than in T2. A significant difference (*p* < 0.05) was observed between the cultivars, with T1 demonstrating a 30.9% higher value than T2. Interestingly, no significant difference between cultivars was detected under moderate and severe salt stress treatments, with T3 being 11.4% higher than T4 and T5 being 4.2% higher than T6. The trend observed for the sodium ion content contrasted that of the potassium ion content, as depicted in Figure 2B. In both cultivars, the sodium ion content increased with increasing salt stress concentration. T3 and T5 exhibited increases of 386% and 473% compared to T1, respectively, while T4 and T6 saw increases of 377% and 456% compared to T2, respectively. Although the sodium ion content of Bai5 was consistently higher than that of Bai2 across all treatments, the difference was not statistically significant. Additionally, the sodium ion content of T4 increased by 20.8% compared to T3, and that of T6 increased by 19.4% compared to T5. As shown in Figure 2C, a decline in calcium ion content was observed within the oat roots alongside an increasing salt stress concentration. Compared to T1, T3 and T5 displayed decreases of 36.1% and 21.98%, respectively. The calcium ion content in T4 and T6 was 5.9% and 45.7% lower than in T2, respectively. Notably, the variation in calcium ion content within oat roots differed between cultivars and treatments; the content in T4 was 24.3% higher than in T3, while the content in T6 was 41.3% lower than in T5. As shown in Figure 2D, the magnesium ion content in oat roots decreased with increasing salt stress concentration. Compared to T1, T3 and T5 exhibited reductions of 4.5% and 6.9%, respectively, while T4 and T6 decreased by 3.9% and 7.5%, respectively, compared to T2. An analysis of magnesium ion content across the two cultivars revealed that Bai2 consistently had higher levels than Bai5. Furthermore, no significant differences were observed among treatments, although T4 displayed a decrease of 1.1% compared to T3, and the magnesium ion content of T6 decreased by 2.3% compared to T5.

### 2.3. Effects of Salt Stress on Oat Root Respiratory Metabolism

**Figure 3:**
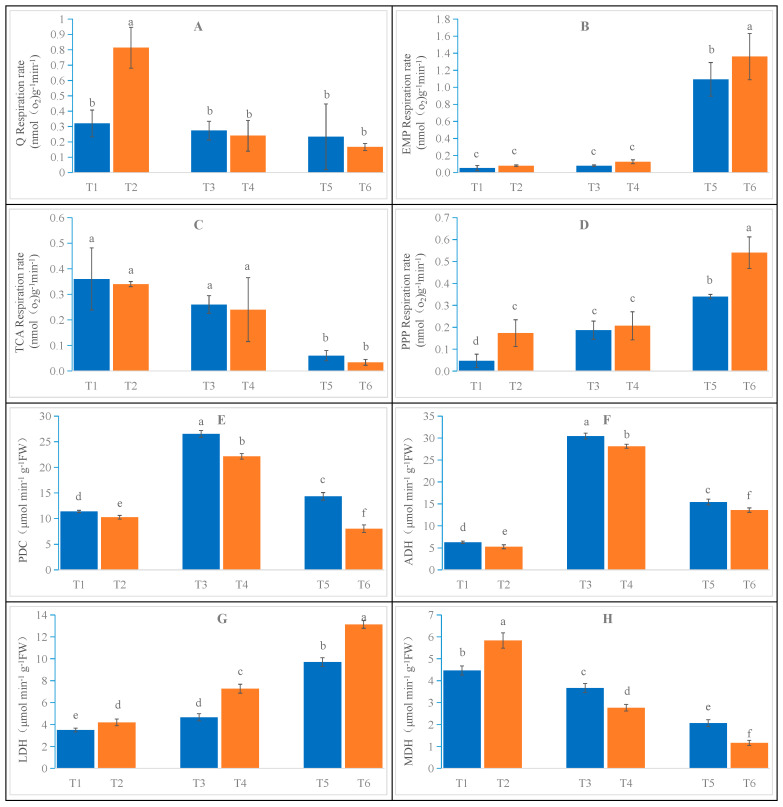
Respiratory metabolism changes in the roots of Bai2 and Bai5 under normal and salt stress conditions. Total respiratory rate (**A**), glycolytic pathway respiratory rate (**B**), tricarboxylic acid cycle pathway respiratory rate (**C**), pentose phosphate pathway respiratory rate (**D**), pyruvate decarboxylase activity (**E**), ethanol dehydrogenase activity (**F**), lactate dehydrogenase activity (**G**), and malate dehydrogenase activity (**H**). Data are the mean ± SD values of three biological replicates (*n* = 3), and different letters indicate significant difference at *p* < 0.05, determined via one-way ANOVA.

Figure 3A demonstrates the impact of salt stress on the total respiration rate of oat root. As the stress concentration increased, inhibition of the root system’s total respiration rate was observed. No significant differences within the same cultivar were detected under varying salt stress concentrations. However, a significant difference was observed between the CK samples of two cultivars. T2 exhibited a 153% increase compared to T1, indicating the presence of inter-varietal differences. Compared to T1, the total respiratory rates of T3 and T5 were inhibited by 15.6% and 28%, respectively. Furthermore, T4 and T6 displayed inhibitions of 70.4% and 79.0%, respectively, compared to T2. Across all salt stress treatments, Bai2 consistently exhibited higher rates than Bai5. Notably, T3 showed a 12.5% increase compared to T4, while T5 demonstrated a 35.3% increase compared to T6.

An increase in salt stress concentration was observed to promote the respiration rate of the oat root glycolysis pathway, as depicted in Figure 3B. However, the overall respiration rate remained higher in Bai5 than in Bai2. Specifically, the glycolytic pathway respiration rates of T3 and T5 increased by 60% and 2080%, respectively, compared to T1. Similarly, T4 and T6 exhibited increases of 62.5% and 1600%, respectively, compared to T2. While T4 showed a 62.5% increase compared to T3, further analysis revealed that Bai2 contributed 29.6% and Bai5 contributed 54.2% to the total respiratory rate. Similarly, T6 displayed a 24.8% increase over T5, with Bai2 contributing 474% and Bai5 contributing 800% to the total respiratory rate.

As the salt stress concentration increased, the respiratory rate of the tricarboxylic acid cycle pathway in oat root was gradually inhibited, as shown in Figure 3C. Under the same stress concentration, no significant difference was observed between the two oat cultivars. Compared to T1, the respiratory rate was inhibited by 27.8% and 83% in T3 and T5, respectively. Similarly, compared to T2, T4 and T6 exhibited inhibitions of 29.4% and 91.2%, respectively. Interestingly, T3 displayed an 8.3% increase compared to T4, with Bai2 contributing 96.3% and Bai5 contributing 100% to the total respiratory rate. Furthermore, T5 demonstrated a 100% increase compared to T6, with Bai2 and Bai5 contributing 26.1% and 17.6% to the total respiratory rate, respectively.

The effect of salt stress on the respiration rate of the pentose phosphate pathway in oat root is shown in Figure 3D. With increasing salt stress concentration, the pentose phosphate pathway’s respiratory rate was progressively stimulated, with Bai5 exhibiting a consistently higher rate than Bai2 across all treatments. Except for the moderate salt stress treatment, significant differences between the cultivar samples that received the two treatments were observed. Compared to T1, T3 and T5 exhibited increases of 280% and 580%, respectively, while T4 and T6 showed increases of 23.5% and 157%, respectively, compared to T2. T4 demonstrated a 10.5% increase relative to T3, with Bai2 contributing 70.4% to the overall respiratory rate and Bai5 contributing 87.5%. T5 displayed a 58.8% increase compared to T6, with Bai2 contributing 148% and Bai5 contributing 318% to the total respiratory rate.

The results presented in Figure 3E indicate that the PDC activity in oat root initially increased and then decreased with increasing salt stress concentration. Notably, Bai2 consistently exhibited higher activity than Bai5 throughout the entire stress period, with significant differences observed between treatments. Compared to T1, T3 and T5 demonstrated increases of 133% and 26%, respectively, while T4 showed an increase of 116.3% compared to T2. In contrast, T6 exhibited a decrease of 21.6% compared to T2. T3 displayed a 19.8% increase over T4, while T5 presented a 78.5% increase compared to T6.

Changes in ADH and PDC activity in the oat roots displayed a consistent trend, as depicted in Figure 3F. Upon increasing the salt stress concentration, ADH activity was activated, initially rising and then declining, with significant differences observed among treatments. Notably, Bai2 exhibited higher activity compared to Bai5. Specifically, T3 and T5 displayed increases of 384% and 145%, respectively, compared to T1. Similarly, T4 and T6 showed increases of 434% and 157%, respectively, compared to T2. Furthermore, T3 exhibited an 8.3% increase compared to T4, while T5 displayed a 13.7% increase compared to T6.

As shown in Figure 3G, a gradual rise in oat root LDH activity was documented with increasing salt stress concentration. Significant differences were observed between the two cultivars across different treatments, with Bai5 demonstrating higher activity than Bai2. Notably, T3 and T5 displayed increases of 33.4% and 177%, respectively, compared to T1. Similarly, T4 and T6 showed increases of 73.1% and 213%, respectively, compared to T2. Moreover, T4 exhibited a 55.7% increase compared to T3, while T6 displayed a 35.4% increase compared to T5. Notably, the increase in LDH activity was greater in Bai5 compared to Bai2.

Figure 3H demonstrates a decrease in oat root MDH activity with increasing salt stress concentration. Except for the control group, where Bai5 exhibited higher enzyme activity than Bai2, all other treatments resulted in higher enzyme activity in Bai2 compared to Bai5, with significant differences observed among treatments. Specifically, T3 and T5 plants displayed decreases of 17.9% and 53.7%, respectively, compared to T1. Similarly, T4 and T6 plants showed decreases of 52.5% and 79.9%, respectively, compared to T2. Furthermore, T4 displayed a 52.5% decrease compared to T3, while T6 exhibited a 43.5% decrease compared to T5.

### 2.4. Effects of Salt Stress on Oat Root Proteomics

**Figure 4:**
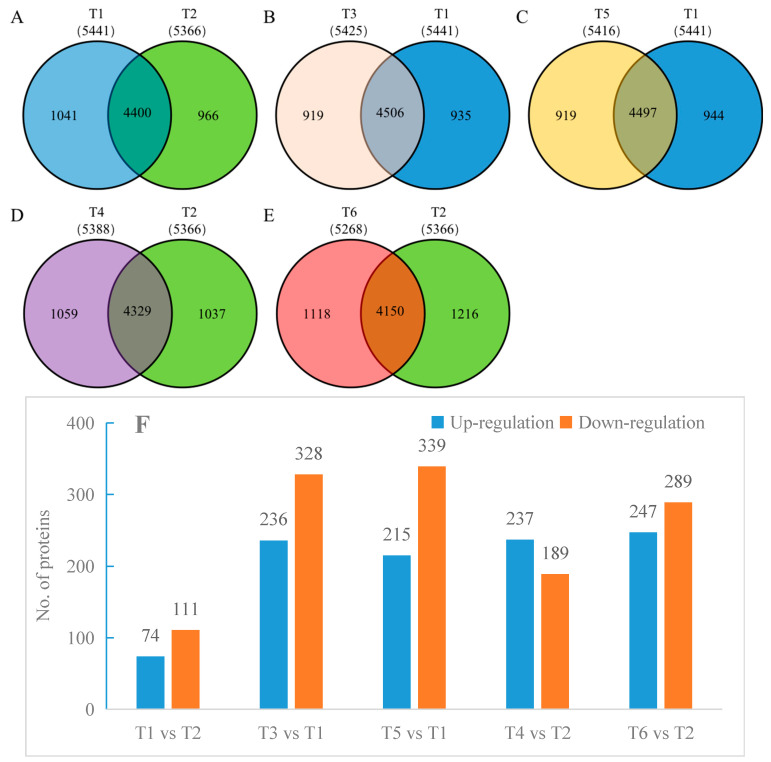
(**A**–**E**) Venn diagrams of total identified and common proteins in the oat root samples, respectively. (**F**) The total number of differentially expressed proteins in different treatment comparisons. Note: When FC ≥ 2.0 and *p*-value ≤ 0.05, screen for upregulated expression proteins, while when FC ≤ 0.50 and *p*-value ≤ 0.05, screen for downregulated expression proteins.

A total of 7174 proteins were identified in the oat roots using label-free proteomics, with all treatments achieving a 99% confidence level. In T1, 5441 proteins were identified; in T2, 5366 proteins were identified; in T3, 5425 proteins were identified; in T4, 5388 proteins were identified; in T5, 5416 proteins were identified; and in T6, 5268 proteins were identified. There were 4400 common proteins identified between T1 and T2 (Figure 4A), 4506 common proteins between T3 and T1 (Figure 4B), 4497 common proteins between T5 and T1 (Figure 4C), 4329 common proteins between T4 and T2 (Figure 4D), and 4150 common proteins between T6 and T2 (Figure 4E). Additionally, based on abundance (FC > 2), 74 proteins were found to be upregulated and 111 proteins downregulated in T1 compared to T2. Between T3 and T1, 236 proteins were upregulated and 328 were downregulated. For T5 versus T1, 215 proteins were upregulated, and 339 were downregulated. Compared to T4, T2 showed 237 upregulated and 189 downregulated proteins. Finally, a comparison of T6 to T2 revealed 247 upregulated and 289 downregulated proteins (Figure 4F).

### 2.5. GO Functional Annotation and Enrichment Analysis of Oat Root Proteome Under Salt Stress

The biological processes impacted by differential proteins between T1 and T2 primarily included the response to stimuli, single-organism processes, metabolic processes, responses to stress, and single-organism metabolic processes. In terms of molecular functions, differences were observed in catalytic activity, electron carrier activity, peroxidase activity, oxidoreductase activity, oxygen binding, transferase activity, isomerase activity, organic cyclic compound binding, heterocyclic compound binding, cofactor binding, and ion binding. The affected cellular components primarily comprised the extracellular space, endoplasmic reticulum membrane, and outer membrane.

**Figure 5:**
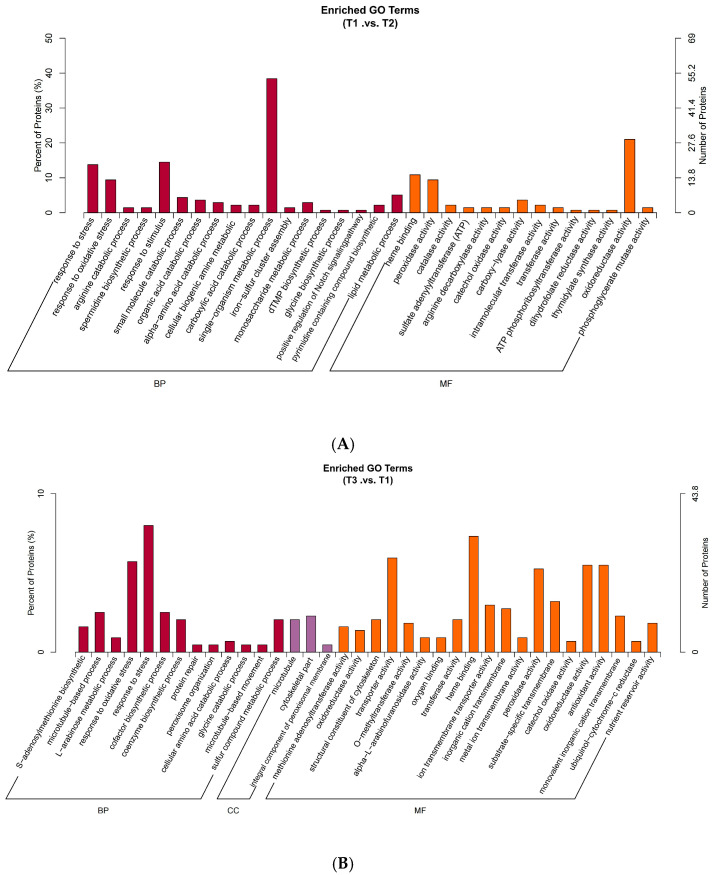

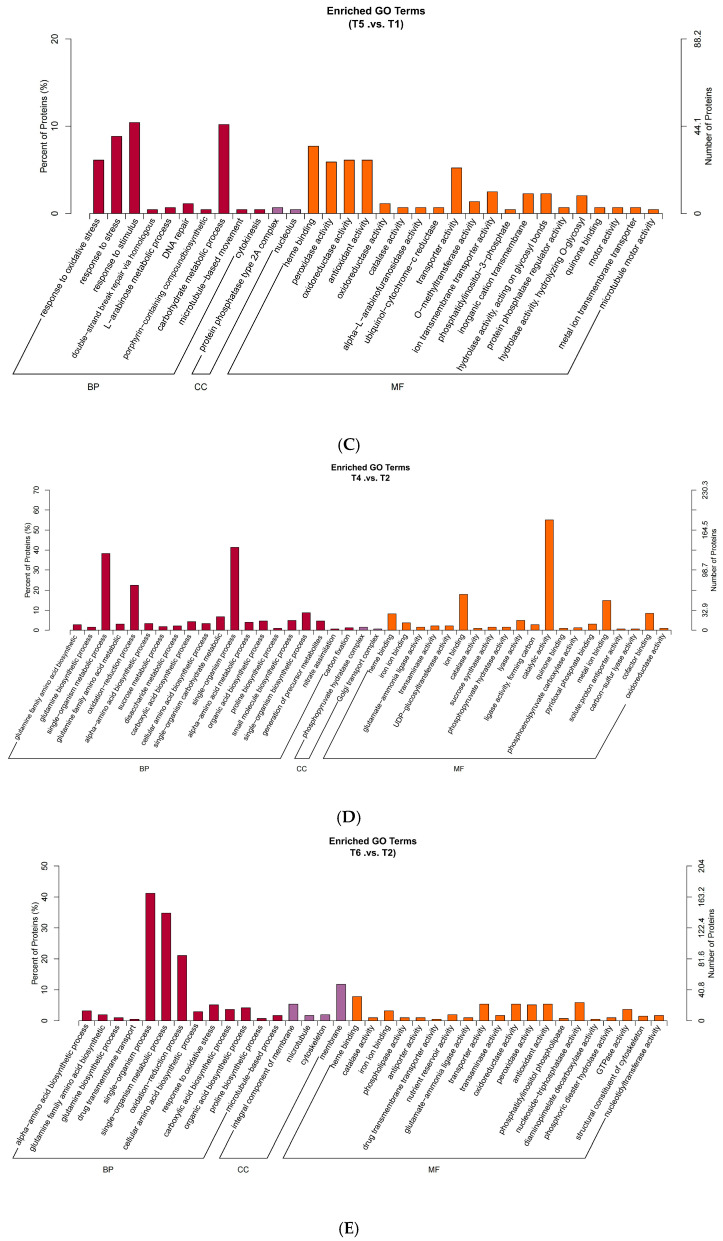
(**A**–**E**) The GO function classification of the DEPs between different treatments in oat root.

When comparing the differential protein responses to salt stress of T3 to T1 and T5 to T1, the main changes detected occurred in molecular functions including heme binding, transporter activity, oxidoreductase activity, oxidizing as acceptor, antioxidant activity, and peroxidase activity. Similarly, a comparison of the differential protein response to salt stress of T4 to T2 revealed that it primarily manifested in molecular functions such as catalytic activity, ion binding, metal ion binding, cofactor binding, and heme binding. Biological processes affected included single-organism processes, single-organism metabolic processes, oxidation–reduction processes, organic acid metabolism processes, single-organism biosynthetic processes, and single-organism carbohydrate metabolic processes. Finally, the differential response of T6 compared to T2 proteins to salt stress was primarily reflected in their molecular functions, like heme binding, nucleoside triphosphate activity, oxidoreductase activity, acting on peroxides as acceptors, antioxidant activity, transporter activity, and peroxidase activity. Single-organism processes, single-organism metabolic processes, oxidation–reduction processes, and responses to oxidative stress were the main biological processes impacted (Figure 5).

### 2.6. KEGG Functional Annotation and Enrichment Analysis of Oat Root Proteome Under Salt Stress

**Figure 6:**
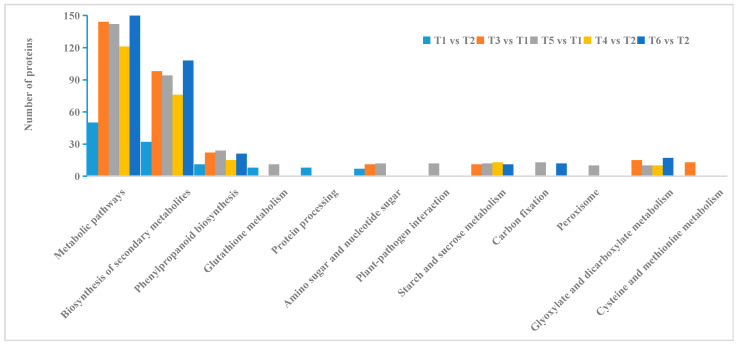
KEGG enrichment analysis results of different treatments in oat root.

Differentially expressed proteins in the root tissues of two oat cultivars under different treatments (Figure 6) underwent KEGG pathway analysis, revealing shared metabolic pathways by comparing differential proteins in the groups, including those related to metabolic pathways, biosynthesis of secondary metabolites, and phenolpropanoid biosynthesis. Additionally, following salt stress, starch and sucrose metabolism and glyoxylate and dicarboxylate metabolism were identified as common metabolic pathways in both cultivars. Specifically, under salt stress, Bai2 exhibited enriched glutathione metabolism, amino sugar and nucleotide sugar metabolism, plant–pathogen interaction, peroxisomes, and cysteine and methionine metabolism pathways. Meanwhile, Bai5 displayed enrichment in carbon metabolism, amino acid biosynthesis, glycolysis/gluconeogenesis, alanine, aspartate and glutamate metabolism, RNA degradation, oxidative phosphorylation, citrate cycle (TCA cycle), and pyruvate metabolism pathways after salt stress.

### 2.7. Screening Differential Proteins

Based on the criteria log2FC > 1 or log2FC < −1, all differentially expressed proteins were screened and classified. This identified a total of 63 differentially expressed proteins within the root of the two oat cultivars (Table S1). These proteins were categorized into various functional groups including, but not limited to, ion transport, protein synthesis, reactive oxygen species (ROS), carbohydrate and energy metabolism, transcriptional regulation and signal transduction, secondary metabolism, and unknown functions. Notably, protein synthesis and carbohydrate and energy metabolism exhibited a high number of differentially expressed proteins.

Furthermore, analysis of the salt-tolerant cultivar Bai2’s root system revealed 111 differentially expressed proteins (Table S2). These proteins fell into twelve functional categories encompassing ribosome, transcription, signal transduction, secondary metabolism, post-translational modification, protein turnover and molecular chaperone, inorganic ion transport and metabolism, carbohydrate and energy metabolism, cytoskeleton and cell wall components, coenzyme transport and metabolism, amino acid transport and metabolism, ROS, and unknown functions. Notably, post-translational modification, protein turnover, and molecular chaperones, along with carbohydrate and energy metabolism, displayed significant differences in protein quantities.

**Figure 7:**
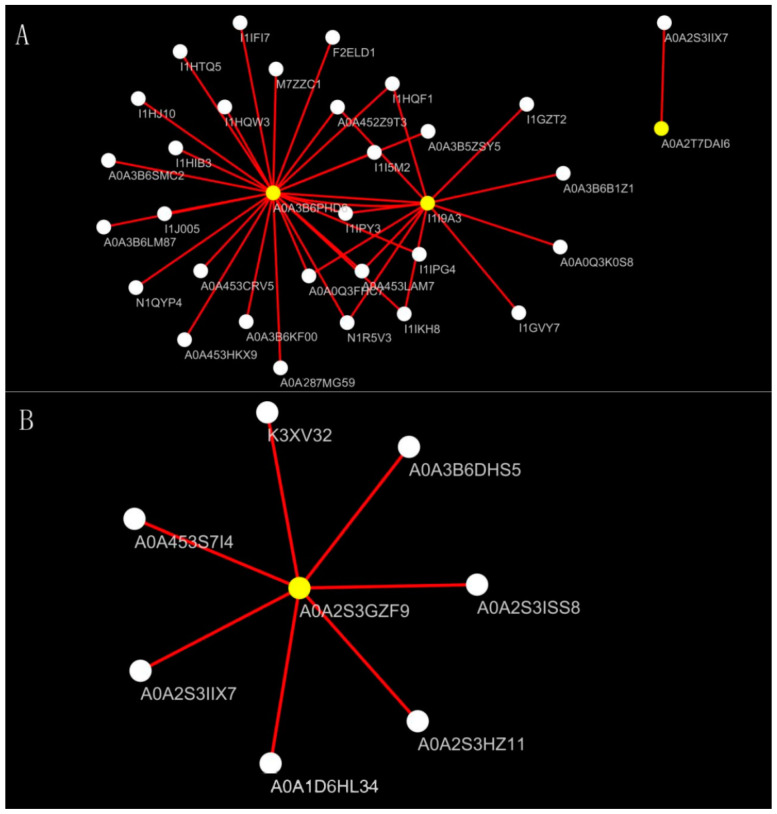

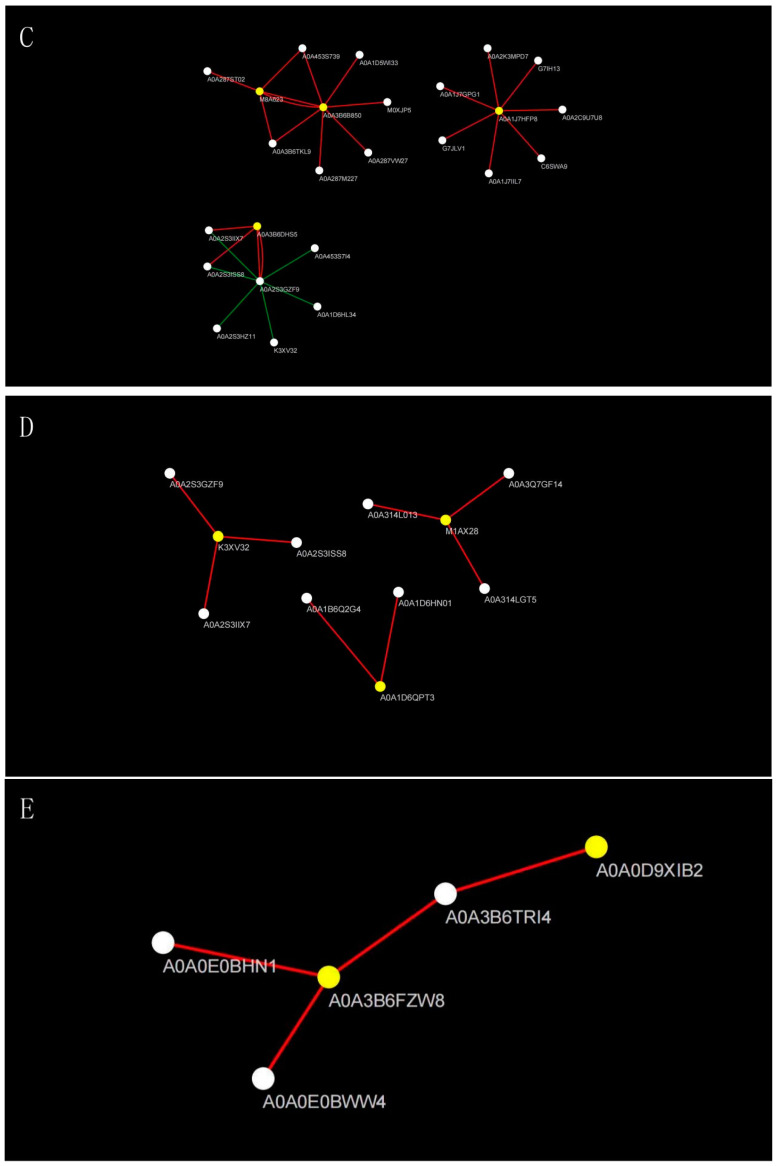
Analysis of differential protein interaction network. Note: Solid lines between nodes indicate protein–protein interactions, with uniform line density and connection strength across the network. Note: (**A**–**E**) represent five metabolic pathways primarily involving antioxidant enzymes, pyruvate metabolism, glycolysis, the tricarboxylic acid cycle and energy metabolism.

The combined action of proteins within a network environment drives their functions in plants. Differential proteins were identified and visualized using the STRING database and Cytoscape software (3.10.0). The analysis focused on differentially expressed proteins in the salt-tolerant cultivar Bai2 under severe salt stress compared to the control group. Interaction network analysis revealed differentially expressed proteins interacting across five metabolic pathways primarily involving antioxidant enzymes (Figure 7A), pyruvate metabolism (Figure 7B), glycolysis (Figure 7C), the tricarboxylic acid cycle (Figure 7D), and energy metabolism (Figure 7E).

Key proteins identified include the following: (1) catalase (A0A3B6PHD6, I1I9A3, A0A2T7DAI6), associated with functions such as signal transduction, carbohydrate metabolism, energy metabolism, and protein synthesis; (2) acetate dehydrogenase (A0A2S3GZF9), associated with protein synthesis, ribosome function, and carbohydrate metabolism; (3) 3-phosphoglycerate kinase (A0A1J7HFP8), glycerol kinase (A0A3B6B850), and aquaporins (M8A623, A0A3B6DHS5), associated with functions such as carbohydrate metabolism, protein synthesis, quantitative metabolism, amino acid transport, and metabolism; (4) phosphoenolpyruvate carboxylase (A0A1D6QPT3, K3XV32, M1AX28), associated with functions such as signal transduction, carbohydrate metabolism, lipid transport, and metabolism; (5) F-type H^+^-transport ATPase subunit-α (A0A3B6FZW8) and V-type H^+^-transATPase subunit-α (A0A0D9XIB2), with functions mainly related to ribosome and protein synthesis.

### 2.8. Fluorescence Quantitative PCR Analysis

**Figure 8:**
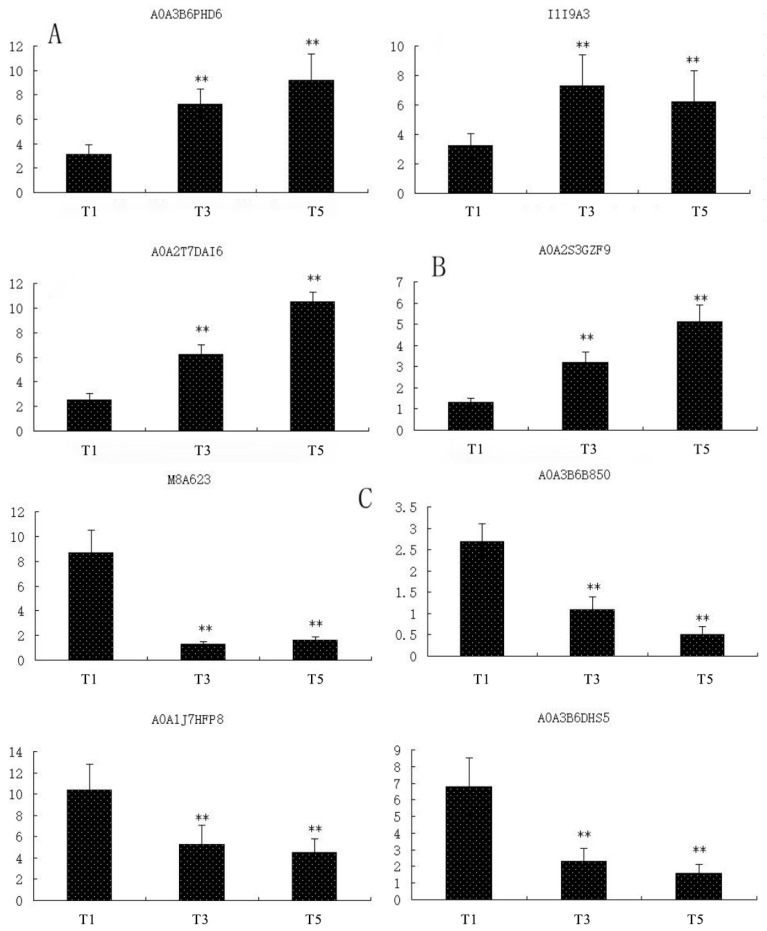

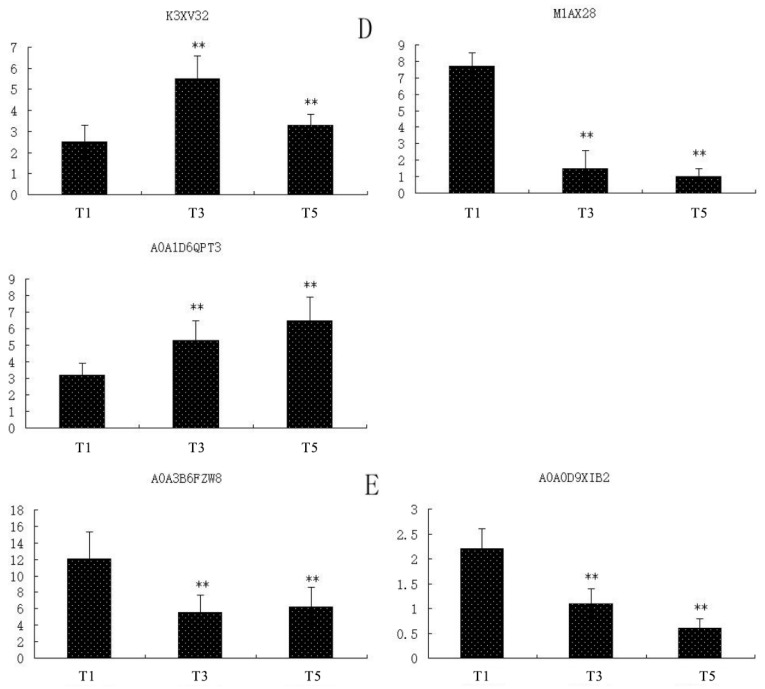
qRT-PCR analysis of differential protein gene expression. Note: Data given in form of mean ± SE; the significant difference is determined by one-way ANOVA (** *p* < 0.01). (**A**–**E**) represent five metabolic pathways primarily involving antioxidant enzymes, pyruvate metabolism, glycolysis, the tricarboxylic acid cycle and energy metabolism.

To examine the expression patterns of related genes, 13 key differential proteins from five pathways of the salt-tolerant oat cultivar Bai2 under severe salt stress were selected for qRT-PCR analysis (Table S3). The expression trends of these genes were consistent with those of their corresponding proteins (Figure 8).

## 3. Discussion

### 3.1. Effects of Salt Stress on Physiological Growth Indicators of Oat

The inhibition of root growth is the most prominent physiological phenomenon observed in plants under salt stress [31]. The root-to-shoot ratio serves as a crucial indicator for assessing seedling quality [32]. Previous studies have demonstrated that a salt concentration of 0.25 mM NaCl or 1.8 dS/m promotes the growth and development of maize seedlings [33]. Conversely, other studies have reported a gradual decrease in the fresh and dry weight of both the aboveground and underground portions of rice with increasing salt concentration [8,34]. In this experiment, the dry and fresh weights of both oat cultivars progressively decreased as the salt stress concentration intensified. The root-to-shoot ratio also exhibited a general downward trend. Notably, the root-to-shoot ratio of Bai2 was lower than that of Bai5 following the control treatment. Furthermore, under identical salt stress concentrations, no significant difference in the root-to-shoot ratio was detected between the two oat cultivars. It is highly conceivable that the insufficient supply of essential sugars, proteins, and other nutrients required for root growth exacerbated the inhibitory effect on root development with the increasing salt stress concentration. Consequently, this led to a decrease in biomass and, in turn, a reduction in the root-to-shoot ratio [35]. As Bai2 exhibited greater salt tolerance compared to Bai5, the decrease in its root-to-shoot ratio with increasing salt stress concentration was less pronounced.

The root system is the first organ to perceive and respond to salt stress, with metabolic alterations directly impacting the plant’s physiological metabolism. The present study showed that under salt stress, the total root length, surface area, volume, and average diameter of oat cultivars were significantly reduced, aligning with established research findings [36]. Indeed, through its effect on ion toxicity, salt stress can hinder plant growth. Na^+^, recognized as the primary toxic ion, can be effectively transported to lessen salt damage [37,38,39]. Previous studies documented an increase in Na^+^ content within the roots and aboveground parts of plants, while the K^+^ content decreased under salt stress [40,41,42,43,44]. This investigation revealed a similar pattern, with the highest Na^+^ content observed in oat roots under severe stress. Notably, the salt-tolerant cultivar, Bai2, maintained a lower Na^+^ content compared to the salt-sensitive Bai5, potentially indicating superior Na^+^ transport capacity under stress conditions. Conversely, salt stress led to reduced K^+^ content in the oat roots, with the salt-tolerant cultivar samples exhibiting higher K^+^ reserves. This might represent a protective mechanism by mitigating K^+^ loss from the plant body. Maintaining K^+^/Na^+^ balance under salt stress is another facet of plant salt tolerance [45]. Significant Na+ uptake under stress can trigger the efflux and loss of K^+^, Ca^2+^, and Mg^2+^ [46]. Notably, the establishment of Ca^2+^ homeostasis within the cytoplasm is crucial for salt adaptation. This experiment consistently observed a gradual reduction in Ca^2+^ content within oat roots under salt stress, mirroring the reported changes in the Ca^2+^ content of wheat [47].

### 3.2. Effects of Salt Stress on Oat Root Respiratory Metabolism

Plants acquire energy for plant growth and development via their respiratory function. Through photosynthesis and respiration, plants convert solar energy into usable forms and store it in their bodies as ATP. This energy fuels their physiological and metabolic activities. Additionally, intermediate products like pyruvate and NADPH, which are generated during respiratory metabolism, are crucial for synthesizing organic substances such as proteins and nucleic acids [35]. Therefore, respiratory metabolism acts as a central energy hub for plants, supplying both energy for life activities and intermediate products for metabolism and synthesis.

Higher plants require sufficient oxygen for respiration and other oxidative pathways to maintain normal growth. Saline–alkali land can cause soil compaction, consequently reducing the oxygen supply and placing considerable pressure on plant growth. This leads to an energy crisis, impacting gene expression; cell metabolism, growth, and development; and, ultimately, biomass [48]. Multiple studies have reported changes in the ratio of carbon dioxide (CO_2_) released to oxygen (O_2_) consumed (respiration quota) in salt-stressed plants. This suggests that mitochondria might be oxidizing different substrates under saline conditions [49,50,51]. In cucumber leaves, salt stress inhibits both the EMP pathway and the TCA cycle, resulting in a decreased respiration rate [52]. Interestingly, some studies have observed an upward trend in the mitochondrial respiration rate of wheat under salt stress in the dark [22]. This study observed that the total root respiration rate of both oat cultivars decreased as the salt stress concentration increased, with a more pronounced decrease in Bai5 compared to Bai2. This difference might contribute to Bai2’s higher salt tolerance. However, the diverse responses observed in different crops under salt stress suggest that stress induces a complex series of coordinated metabolic changes involving numerous pathways, necessitating further investigation.

Three respiratory metabolic pathways exist in plants: the glycolytic, tricarboxylic acid cycle, and pentose phosphate pathways. Under normal conditions, the TCA pathway predominantly sustains plants. Studies have demonstrated that temperature stress alters the proportion of respiratory pathways in cotton seedlings, with changes in the PPP closely associated with adverse growth environments [53]. In this study, increasing salt stress concentrations progressively inhibited the TCA pathway while simultaneously enhancing the EMP pathway and PPP. This may result from salt stress-induced insufficiencies in metabolite supply, ATP energy, and reducing NADH, thereby hindering normal cell growth. Inhibition of the TCA pathway obstructs the entry of pyruvate, an oxidative degradation product from the EMP pathway, into the TCA cycle. Consequently, the PPP becomes activated, gradually replacing the TCA pathway as the primary biochemical respiratory pathway [54].

The present study’s findings revealed that salt stress promotes the anaerobic metabolic pathway in oat root. During anaerobic respiration, pyruvate generated by glycolysis is converted into lactic acid under the influence of LDH; it is then further converted into ethanol under the combined action of ADH and pyruvate dehydrogenase. This process ensures the smooth operation of the EMP pathway by oxidizing NADH to NAD+, providing ATP for anaerobic respiration [55]. This aligns with numerous research findings, suggesting that enhanced anaerobic respiration metabolism in oat root and increased PDC, ADH, and LDH activities can improve plant tolerance to salt stress [56,57]. Under salt stress, the PDC and ADH enzyme activities in the roots of both oat cultivars exhibited an initial increase followed by decrease. However, overall, the salt-tolerant cultivar Bai2 displayed a higher level of enzyme activity compared to the salt-sensitive cultivar Bai5, indicating its primary reliance on pyruvate ethanol fermentation for anaerobic respiration energy supply. Conversely, while LDH enzyme activity in Bai5 continued to increase with increasing salt stress, it remained higher than in Bai2 overall. This suggests that Bai5 primarily relies on pyruvate lactate fermentation for anaerobic respiration energy, and the significant increase in lactate metabolism leads to substantial lactate accumulation in the oat plant, resulting in cytoplasmic acidification and ultimately reduced salt stress tolerance. Due to the design of this experiment and soil conditions, it is unclear whether the effect of salt stress on oat root respiration metabolism is caused by salt toxicity, low oxygen conditions due to salt soil compaction, or both. It is necessary to solve this problem through hydroponic experiments or more detailed experimental designs in the future.

### 3.3. Effects of Salt Stress on Oat Root Proteomics

**Figure 9:**
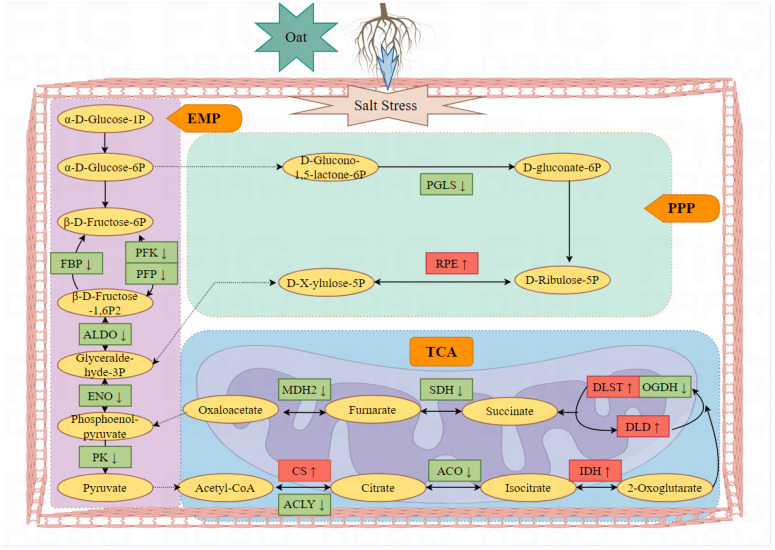
Schematic diagram of key respiratory metabolism response proteins of salt-tolerant cultivar Bai2 in response to salt stress (figure drawn using Figdraw 2.0).

Under salt stress conditions, a total of 7174 proteins were analyzed in the roots of two oat cultivars, resulting in the identification of differentially expressed proteins spanning multiple functional categories. Following an interaction network analysis and the categorization of main functions, it was revealed that the majority of differentially expressed proteins participate in protein synthesis, carbohydrate and energy metabolism, redox processes, and other functions (Figure 9).

Plants are known to adapt to stress by maintaining a protein balance, with ribosomes playing a critical role in regulating cell growth and development through protein synthesis [58]. This study identified a substantial number of differentially expressed proteins associated with protein synthesis, leading to the conclusion that it represents a key metabolic process used by oat seedling root systems to cope with salt stress. These differentially expressed proteins primarily included various ribosomal proteins, translation initiation factors, and peptide chain release factors. Notably, most of these proteins were downregulated under salt stress, suggesting a decrease in overall protein synthesis in oat seedling roots, and consequently, in their growth rate. This observation aligns with existing research on kidney beans and suggests an adaptation involving reduced water consumption [59]. Notably, the upregulation of some proteins involved in protein synthesis highlights the complexity of this process [60].

Carbohydrates, which are widely present in plants, are both products of photosynthetic assimilation and substrates of respiration [61]. This study focused on carbohydrate and energy metabolism processes, primarily glycolysis and the tricarboxylic acid cycle. Glycolysis involves breaking down glucose in plants to produce energy. Network interaction correlation and verification resulted in the identification of the main proteins related to glycolysis (A0A1J7HFP8, A0A3B6DHS5, A0A3B6B850, and M8A623), all of which were downregulated under salt stress conditions. Pyruvate dehydrogenase, which is crucial in glycolysis for catalyzing the oxidative decarboxylation of pyruvate to acetyl CoA and NADH, is also a key factor for glycolysis entering the tricarboxylic acid cycle [62]. The upregulation of this enzyme suggests a stronger respiratory function and capacity for energy production in Bai2 under severe salt stress. Indeed, it is widely acknowledged that cellular metabolism primarily relies on the tricarboxylic acid cycle pathway, which is essential for plant life activities and provides significantly more energy compared to glycolysis. Among the three differentially expressed proteins involved in this pathway, only A0A1D6QPT3 was upregulated and the other two were downregulated, indicating inhibited respiration in oat roots under salt stress.

When subjected to abiotic stress, elevated reactive oxygen species production in plants hinders their growth [63,64]. Consequently, ROS clearance becomes a crucial mechanism for plant tolerance to abiotic stress. Antioxidases, such as superoxide dismutase (SOD), peroxidase (POD), and catalase (CAT), are a class of differentially expressed proteins involved in ROS clearance. The identified PODs (J3MP40, M7ZH16, A0A077RUR2, A0A0J8BPV6, A0A2T7DXQ7, A0A3B6RDG1, and I1HF19) were all downregulated following salt stress, suggesting their increased sensitivity to this stress. Conversely, CAT (A0A0Q3F350, A0A3B6PHD6, I1I9A3, and A0A2T7DAI6) was upregulated after salt stress, with a more pronounced upregulation in Bai5. Extensive studies in plants like Arabidopsis, rice, wheat, and rapeseed have demonstrated the involvement of proteins like POD, SOD, and CAT in stress responses [65,66,67]. The findings of this study support the presence of multiple ROS clearance mechanisms contributing to salt tolerance in oat.

## 4. Materials and Methods

### 4.1. Overview of Experimental Site

This experiment was conducted in the greenhouse of the Oat Industry Research Center of Inner Mongolia Agricultural University. Greenhouse culture conditions included 16 h light at 25–30 °C and 8 h of darkness at 15–18 °C, with natural light and plant supplementary light supplement. Oat plants were grown in plastic barrels with an upper diameter of 24 cm, a lower diameter of 22 cm, and a height of 25 cm. The substrate was taken from the 0–20 cm topsoil layer of a field, with a soil capacity of 6.5 kg per pot. The basic soil nutrients are shown in Table 4.

### 4.2. Test Materials

The two tested cultivars, the salt-tolerant cultivar Bai2 and salt-sensitive cultivar Bai5, were selected and bred by the Baicheng Academy of Agricultural Sciences in Jilin Province [68].

### 4.3. Experimental Design

The salt components used in the test were NaCl and Na_2_SO_4_ mixed in a 1:1 molar ratio. Three salt stress concentrations were established: control (0 mM), moderate stress (100 mM), and severe stress (150 mM). The two oat cultivars, Bai2 and Bai5, were subjected to these treatments, resulting in a total of six treatment combinations. Each combination was replicated 3 times with 4 pots per replicate for a total of 72 pots. Prior to seeding, 150 kg·hm^−2^ of diammonium phosphate fertilizer was applied, and 1.5 L of salt stress solution (100 mM or 150 mM) was added to the pot. Control pots received an equal volume of water. When the soil moisture content was suitable, 40 seeds were planted per pot. Every 3 days, 250 mL of hydration was provided. The indexes were determined 3 days after the emergence of oat seedlings.

### 4.4. Determination Indexes and Methods

#### 4.4.1. Determination of Biomass and Root–Shoot Ratio of Oat Seedlings

On the day of sampling, five representative plants were selected from each treatment. Their root substrate was rinsed with tap water, and surface moisture was absorbed with filter paper. The fresh weight of both the aboveground and underground parts was measured. For the aboveground parts, the fresh weight was recorded individually for each plant. After numbering, they were placed in labeled envelopes. To inactivate enzymes, the green parts were subjected to a temperature of 105 °C for 15 min, followed by drying at 80 °C until a constant weight was achieved. The dry weight was measured for each plant, and the average value for each treatment was calculated to obtain the aboveground and underground biomass.

#### 4.4.2. Determination of Root Morphological Indexes

Three representative plants from each treatment and replicate were selected. Their root systems were scanned and stored on a computer using a digital scanner (Epson V700, Beijing, China). WinRhizo PRO 2013 root analysis system software (Regent Instruments, Quebec, QC, Canada) was used for quantitative analysis, yielding data such as the total root length, surface area, volume, and mean diameter.

#### 4.4.3. Determination of Root Ion Content

Dried roots from both Bai2 and Bai5 were digested using a HNO_3_ acid solution in a microwave digestion instrument (Multiwave 3000, Anton Paar GmbH, North Ryde, Australia). We determined the sodium, potassium, calcium, and magnesium contents in the digested solution using an inductively coupled plasma–optical emission spectrometer (iCAP 6000 series, Thermo Fisher scientific, Waltham, MA, USA), as per the manufacturer’s instructions. Using the SPSS software (IBM SPSS Statistics Version 19.0), we performed a one-way ANOVA of associations of different element concentrations between the treatments, where *p* < 0.05 and 0.01 were considered markedly significant and highly significant, respectively.

#### 4.4.4. Determination of Root Respiration Rate

The root respiration rate was measured as the oxygen consumption rate using an Oxytherm oxygen electrode (Hansatech, King’s Lynn, Norfolk, UK). Fresh root samples (500 mg) from each replicate were used for the measurements. NaF, malonic acid, Na_3_PO_4_, hydroxyzine salicylic acid, and sodium cyanide were employed as inhibitors of the Embden–Meyerhof pathway (EMP), tricarboxylic acid cycle (TCA), and pentose phosphate pathway (PPP), respectively. The difference in oxygen consumption between root treated with and without a specific inhibitor was considered to represent the respiration rate of the corresponding pathway.

Basic biochemical pathways and the total respiratory rate (Q) were determined using Oxytherm liquid-phase oxygen electrodes (Chlorolab-2, Hansatech Scientific Instruments, Pentney, UK). Three plants per treatment were sampled, and their roots were immediately washed and stored in a 5 mM MES (pH of 5.5; 1 mM CaSO_4_) buffer solution for testing. Fresh root samples (0.1 g) were weighed, cut into pieces, and placed in the instrument’s reaction cup. The root respiration rate was expressed as the amount of oxygen consumed per unit fresh weight per unit time.

Specific respiratory pathways were determined using the Yu Rangcai method. The following specific inhibitors were employed: 10 mM NaF (enolase, specifically inhibiting the EMP pathway), 50 mM malonic acid (specifically inhibiting the tricarboxylic acid cycle), and 10 mM Na_3_PO_4_ (specifically inhibiting the pentose phosphate pathway). Each inhibitor was prepared in 0.05 M phosphate buffer (pH 6.5).

#### 4.4.5. Measurement of Enzyme Activity Related to Respiratory Metabolism

1. Pyruvate decarboxylase activity determination

A fresh oat root sample (0.1 g) was weighed and homogenized thoroughly with 1 mL of Tris buffer on ice. The homogenate was then centrifuged at 16,000× *g* for 20 min at 4 °C. The supernatant was collected and kept on ice for measurement. The assay was performed according to the instructions provided by Shanghai Qiyi Biotechnology Co., Ltd. (Shanghai, China), with colorimetric measurements at 340 nm. The absorbance values were recorded at 15 and 75 s, and the change in absorbance (ΔA) was calculated as ΔA = A15 − A75.

2. Determination of alcohol dehydrogenase activity

Samples were pretreated with PDC. Reagents were added in accordance with the steps provided by Shanghai Qiyi Biotechnology Co., Ltd. After zeroing the instrument with distilled water, colorimetric readings were taken at 340 nm. Absorption values at 15 s and 75 s were recorded, and the difference (ΔA) was calculated as A15 − A75.

3. Determination of lactate dehydrogenase (LDH) activity

A fresh oat root sample (0.1 g) was weighed and combined with 1 mL of extract solution. The mixture was fully homogenized under ice bath conditions before it was centrifuged at 4 °C and 8000× *g* for 10 min. The supernatant was then collected and kept on ice for measurement. Following the instructions provided in the kit from Shanghai Qiyi Biotechnology Co., Ltd., the reagent was added, mixed thoroughly, and incubated at room temperature for 3 min. The absorbance of each tube was determined at 450 nm using distilled water as the blank. Finally, the change in absorbance (ΔA) was calculated as A(determination tube) − A(CK).

4. Determination of malate dehydrogenase (NAD-MDH) activity

A 0.1 g sample of fresh oat root was weighed. Then, 1 mL of extract was added, and the mixture was fully ground into a homogenate under ice bath conditions. It was then centrifuged at 4 °C and 8000× *g* for 10 min. The supernatant was collected and placed on ice for further measurement. Following the kit instructions provided by Shanghai Qiyi Biotechnology Co., Ltd., the reagent was added, and both the initial absorbance value (A1) and the absorbance value (A2) after 1 min of reaction at 340 nm wavelength were immediately recorded. Finally, the change in absorbance (ΔA) was calculated using the formula ΔA = A1 − A2.

#### 4.4.6. Root Proteome Determination and Methods

1. Root pretreatment

For each treatment, fresh oat root samples were taken. They were washed with tap water, rinsed with deionized water 3–5 times, and dried with filter paper. They were then loaded into 2 mL frozen tubes, quickly frozen with liquid nitrogen, and stored at −80 °C for measurement. Three oat roots were used as a biological replicate for each treatment, with a total of three replicates.

2. Extraction of total protein from oat root

Frozen samples intended for measurement were crushed using a liquid nitrogen precooled crusher. The resulting powder was further ground with liquid nitrogen to a fine consistency. Lysis buffer (100 mmol·L^−1^ NH_4_HCO_3_ + 6 mol·L^−1^ CH_4_N_2_O + 0.2% SDS, pH = 8) was added to the powder in a 1:10 (*w/v*) ratio and mixed thoroughly. Ultrasonication was carried out at an amplitude of 22% with on cycles lasting 0.2 s and 2 s off cycles for a total of 60 s. The mixture was then extracted at room temperature for 30 min. Subsequently, centrifugation was performed at 15,000× *g* for 1 h at 10 °C. The supernatant was collected, aliquoted, and frozen at −80 °C.

3. Protein quantification

Protein concentrations were determined using the Bradford method. The protein concentration of each sample was calculated according to the curve formula (μg·μL^−1^).

4. Filter-aided sample preparation (FASP)

Following protein quantification, 200 μg of protein solution was aliquoted into a centrifuge tube. Dithiothreitol (DTT) was added to achieve a final concentration of 25 mM, and the reaction was incubated at 60 °C for 1 h. Iodoacetamide was then added to reach a final concentration of 50 mM, and the mixture was incubated at room temperature for 10 min. The reduced and alkylated protein solution was subsequently transferred to a 10 kDa ultrafiltration tube and centrifuged at 12,000× *g* for 20 min. The solution at the bottom of the collection tube was discarded. A total of 100 μL of issolution buffer (6 mol·L^−1^ CH_4_N_2_O + 100 mmol·L^−1^ TEAB, pH = 8.5) was added, followed by centrifugation at 12,000× *g* for 20 min. The solution at the bottom was again discarded, and this step was repeated three times. A new collection tube was employed. Moreover, 1 μg·μL^−1^ trypsin 3 μL and 100 mmol·L^−1^ TEAB buffer 500 μL were added to the ultrafiltration tube. The reaction was allowed to proceed overnight at 37 °C. The following day, the mixture was centrifuged at 12,000× *g* for 20 min, and the digested peptide solution at the bottom of the tube was collected. Another 50 μL of dissolution buffer was added to the ultrafiltration tube, followed by centrifugation at 12,000× *g* for 20 min. The resulting supernatant was combined with the previously collected peptide solution, yielding a total volume of 100 μL of enzymatically hydrolyzed sample. This sample was then freeze-dried for further analysis.

5. Nanoflow reversed-phase chromatography-Q Exactive for protein analysis

A solution of 20 μL containing 2% methanol and 0.1% formic acid was prepared for this experiment. The solution was centrifuged at 12,000× *g* for 10 min, and the supernatant was collected for sample loading. A sample volume of 10 μL was loaded using a loading pump with a flow rate of 350 nL·min^−1^ for 15 min, while the separation flow rate was 300 nL·min^−1^.

6. Mass spectrum data analysis

The database uniprot-Pooideae361804_20170619.fasta.fasta (362,934 sequences) was utilized for mass spectrometry analysis, which was conducted using a Thermo Q Exactive mass spectrometer (Thermo Fisher scientific, Waltham, MA, USA). Peptide Spectrum Matches (PSMs) with confidence levels exceeding 95% were deemed trusted PSMs. Proteins containing at least one unique peptide segment (a specific peptide segment) were considered trusted proteins. Only credible peptides and proteins were retained, and FDR verification was carried out to eliminate those with an FDR greater than 1%. For comparisons between pairs of samples, the mean of protein difference multiples in different replication groups was employed as the difference multiple for those samples. Significance was assessed using the *t*-test, yielding *p*-values.

### 4.5. Data Processing and Bioinformatics Analysis

Microsoft Excel 2010 was used for data calculation and processing and generating charts, and SAS 9.0 software was used for significance analysis (*p* < 0.05). We performed one-way ANOVA analysis using the software SPSS (IBM SPSS Statistics Version 19.0) to associate different physiological indexes between the treatments. The identified proteins were annotated using common functional databases, including COG, GO, and KEGG databases. Finally, the differential proteins underwent functional analysis to identify significantly enriched GO terms and KEGG pathways.

## 5. Conclusions

This study investigated the response mechanisms of different salt-tolerant oat cultivars to salt stress. Label-free technology was employed to quantitatively analyze 18 samples of oat root from both cultivars. We identified candidate differentially expressed proteins and explored key metabolic pathways to provide new references for future research. Interaction network analysis revealed that at a concentration of 300 mM, five differentially expressed proteins interacting with metabolic pathways were detected in Bai2. These pathways primarily involve antioxidant enzymes, pyruvate metabolism, glycolysis, the tricarboxylic acid cycle, and energy metabolism. In the following research, their functions will be validated through genetic modification and other methods, and the research results will be applied to molecular breeding of crops such as oat. By using Oxytherm liquid-phase oxygen electrode, we found that Bai2 primarily utilizes pyruvate ethanol fermentation for its anaerobic respiration energy supply, while Bai5 relies mainly on pyruvate lactate fermentation for the same purpose. This significant discovery revealed for the first time from the perspective of respiratory metabolism that different salt-tolerant oat cultivars adapt to salt stress in different ways to maintain normal growth and development. The experimental results provide new insights into plant adaptation to salt stress from the perspective of respiratory metabolism. The experimental results provide new insights into plant adaptation to salt stress from the perspective of respiratory metabolism.

## Figures and Tables

**Table 1:** Treatment codes of two oat cultivars under salt stress at different concentrations.

	Treatment	0 mM	100 mM	150 mM
Cultivar				
Bai2		T1	T3	T5
Bai5		T2	T4	T6

**Table 2:** Effects of salt stress on the freshness, dry weight, and root–shoot ratio of oat.

Treatment	Fresh Weight (g)		Dry Weight (g)		Root–Shoot Ratio
	Aboveground	Underground	Aboveground	Underground	
T1	0.445 ± 0.09 ^ab^	0.110 ± 0.02 ^ab^	0.047 ± 0.01 ^ab^	0.008 ± 0.00 ^b^	0.182 ± 0.03 ^ab^
T2	0.502 ± 0.07 ^a^	0.166 ± 0.08 ^a^	0.053 ± 0.01 ^a^	0.014 ± 0.01 ^a^	0.257 ± 0.10 ^ab^
T3	0.373 ± 0.11 ^abc^	0.060 ± 0.03 ^bc^	0.048 ± 0.01 ^ab^	0.004 ± 0.00 ^b^	0.083 ± 0.03 ^c^
T4	0.323 ± 0.05 ^bc^	0.042 ± 0.01 ^c^	0.046 ± 0.01 ^ab^	0.005 ± 0.00 ^b^	0.108 ± 0.00 ^bc^
T5	0.343 ± 0.02 ^bc^	0.055 ± 0.01 ^bc^	0.044 ± 0.00 ^ab^	0.005 ± 0.00 ^b^	0.124 ± 0.04 ^bc^
T6	0.268 ± 0.08 ^c^	0.020 ± 0.01 ^c^	0.036 ± 0.00 ^b^	0.004 ± 0.00 ^b^	0.112 ± 0.02 ^bc^

Note: different lowercase letters indicate significant differences between two cultivars and three salt concentrations (*p* < 0.05).

**Table 3:** Effects of salt stress on oat root morphology.

Treatment	Length (cm)	Surface Area (cm^2^)	Volume (cm^3^)	Average Diameter (mm)
T1	224.53 ± 6.46 ^a^	41.43 ± 1.20 ^a^	1.47 ± 0.06 ^a^	0.62 ± 0.00 ^a^
T2	209.17 ± 2.85 ^b^	36.92 ± 0.44 ^b^	1.27 ± 0.03 ^b^	0.61 ± 0.00 ^ab^
T3	184.97 ± 4.90 ^c^	31.57 ± 1.58 ^c^	1.06 ± 0.07 ^c^	0.60 ± 0.02 ^b^
T4	162.20 ± 4.03 ^d^	26.90 ± 0.48 ^d^	0.77 ± 0.04 ^d^	0.57 ± 0.00 ^c^
T5	142.33 ± 5.22 ^e^	23.69 ± 1.36 ^e^	0.56 ± 0.04 ^e^	0.56 ± 0.00 ^c^
T6	124.20 ± 9.00 ^f^	20.45 ± 0.71 ^f^	0.32 ± 0.01 ^f^	0.54 ± 0.00 ^d^

Note: different lowercase letters indicate significant differences between two cultivars and three salt concentrations (*p* < 0.05).

**Table 4:** Basic nutrients of potted soil.

Soil Type	pH	Salt Content (g·kg^−1^)	Organic Matter (g·kg^−1^)	Alkaline Hydrolysis of Nitrogen (mg·kg^−1^)	Available Phosphorus (mg·kg^−1^)	Quick-Acting Potassium (mg·kg^−1^)
Clay	7.81	0.82	20.60	55.18	1.61	145.60

## Data Availability

Data are contained within the article and its Supplementary Materials. Proteomic data can be found in the Oat Base database (http://112.126.28.109:8086, accessed on 15 January 2025).

## Supplementary Materials

The supporting information can be downloaded at: https://www.mdpi.com/article/10.3390/ijms26062630/s1.

## References

[1] Mondal S., Borromeo T.H. (2016). Screening of Salinity Tolerance of Rice at Early Seedling Stage. J. Biosci. Agric. Res..

[2] Qadir M., Quillérou E., Nangia V., Murtaza G., Singh M., Thomas R.J., Drechsel P., Noble A.D. (2014). Economics of Salt-induced Land Degradation and Restoration. Proceedings of the Natural Resources Forum.

[3] Pessarakli M., Szabolcs I. (2019). Soil Salinity and Sodicity as Particular Plant/Crop Stress Factors. Handbook of Plant and Crop Stress.

[4] Nam T.N., Thia L.H., Mai D.S., Tuan N.V. (2017). Overexpression of NbWRKY79 Enhances Salt Stress Tolerance in Nicotiana Benthamiana. Acta Physiol. Plant..

[5] Wang B., Song F.B. (2006). Physiological Responses and Adaptive Capacity of Oats to Saline-Alkali Stress. Ecol. Environ..

[6] Jia X., Wang H., Svetla S., Zhu Y., Hu Y., Cheng L., Zhao T., Wang Y. (2019). Comparative Physiological Responses and Adaptive Strategies of Apple Malus Halliana to Salt, Alkali and Saline-Alkali Stress. Sci. Hortic..

[7] Kumari A., Das P., Parida A.K., Agarwal P.K. (2015). Proteomics, Metabolomics, and Ionomics Perspectives of Salinity Tolerance in Halophytes. Front. Plant Sci..

[8] Hussain S., Zhang J., Zhong C., Zhu L., Cao X., Yu S., Bohr J.A., Hu J., Jin Q. (2017). Effects of Salt Stress on Rice Growth, Development Characteristics, and the Regulating Ways: A Review. J. Integr. Agric..

[9] McCormack M.L., Dickie I.A., Eissenstat D.M., Fahey T.J., Fernandez C.W., Guo D., Helmisaari H., Hobbie E.A., Iversen C.M., Jackson R.B. (2015). Redefining Fine Roots Improves Understanding of Below-ground Contributions to Terrestrial Biosphere Processes. New Phytol..

[10] Van Dongen J.T., Gupta K.J., Ramírez-Aguilar S.J., Araújo W.L., Nunes-Nesi A., Fernie A.R. (2011). Regulation of Respiration in Plants: A Role for Alternative Metabolic Pathways. J. Plant Physiol..

[11] Li L., Lv F., Guo Y., Wang Z. (2016). Respiratory Pathway Metabolism and Energy Metabolism Associated with Senescence in Postharvest *Broccoli* (*Brassica oleracea* L. Var. Italic style) Florets in Response to O_2_/CO_2_ Controlled Atmospheres. Postharvest Biol. Technol..

[12] Buchanan B.B., Balmer Y. (2005). Redox Regulation: A Broadening Horizon. Annu. Rev. Plant Biol..

[13] Tao S., Zhu Y., Pan Y., Zhang Z., Huang L. (2022). Enhancement of Respiratory Metabolism of the Pentose Phosphate Pathway (PPP) Strengthens the Chilling Tolerance of Postharvest Papaya Fruit Stored at 1 °C. Postharvest Biol. Technol..

[14] Shu L., Lou Q., Ma C., Ding W., Zhou J., Wu J., Feng F., Lu X., Luo L., Xu G. (2011). Genetic, Proteomic and Metabolic Analysis of the Regulation of Energy Storage in Rice Seedlings in Response to Drought. Proteomics.

[15] Fukao T., Xu K., Ronald P.C., Bailey-Serres J. (2006). A Variable Cluster of Ethylene Response Factor–like Genes Regulates Metabolic and Developmental Acclimation Responses to Submergence in Rice. Plant Cell.

[16] Mustroph A., Barding G.A., Kaiser K.A., Larive C.K., Bailey-Serres J. (2014). Characterization of Distinct Root and Shoot Responses to Low-oxygen Stress in A Rabidopsis with a Focus on Primary C-and N-metabolism. Plant Cell Environ..

[17] Weits D.A., Kunkowska A.B., Kamps N.C.W., Portz K.M.S., Packbier N.K., Nemec Venza Z., Gaillochet C., Lohmann J.U., Pedersen O., van Dongen J.T. (2019). An Apical Hypoxic Niche Sets the Pace of Shoot Meristem Activity. Nature.

[18] Rocha M., Licausi F., Araújo W.L., Nunes-Nesi A., Sodek L., Fernie A.R., Van Dongen J.T. (2010). Glycolysis and the Tricarboxylic Acid Cycle Are Linked by Alanine Aminotransferase during Hypoxia Induced by Waterlogging of Lotus Japonicus. Plant Physiol..

[19] Zhong M., Yuan Y., Shu S., Sun J., Guo S., Yuan R., Tang Y. (2016). Effects of Exogenous Putrescine on Glycolysis and Krebs Cycle Metabolism in Cucumber Leaves Subjected to Salt Stress. Plant Growth Regul..

[20] Jacoby R.P., Taylor N.L., Millar A.H. (2011). The Role of Mitochondrial Respiration in Salinity Tolerance. Trends Plant Sci..

[21] Li L., Xing W., Shao Q., Shu S., Sun J., Guo S. (2015). The Effects of Grafting on Glycolysis and the Tricarboxylic Acid Cycle in Ca (NO_3_)_2_-Stressed Cucumber Seedlings with Pumpkin as Rootstock. Acta Physiol. Plant..

[22] Che-Othman M.H., Jacoby R.P., Millar A.H., Taylor N.L. (2020). Wheat Mitochondrial Respiration Shifts from the Tricarboxylic Acid Cycle to the GABA Shunt under Salt Stress. New Phytol..

[23] Kato-Noguchi H., Morokuma M. (2007). Ethanolic Fermentation and Anoxia Tolerance in Four Rice Cultivars. J. Plant Physiol..

[24] Kennedy R.A., Rumpho M.E., Fox T.C. (1992). Anaerobic Metabolism in Plants. Plant Physiol..

[25] Rahman M., Grover A., Peacock W.J., Dennis E.S., Ellis M.H. (2001). Effects of Manipulation of Pyruvate Decarboxylase and Alcohol Dehydrogenase Levels on the Submergence Tolerance of Rice. Funct. Plant Biol..

[26] Ismond K.P., Dolferus R., De Pauw M., Dennis E.S., Good A.G. (2003). Enhanced Low Oxygen Survival in Arabidopsis through Increased Metabolic Flux in the Fermentative Pathway. Plant Physiol..

[27] Kursteiner O., Dupuis I., Kuhlemeier C. (2003). The Pyruvate Decarboxylase1 Gene of Arabidopsis Is Required during Anoxia but Not Other Environmental Stresses. Plant Physiol..

[28] Richardson S.G., McCree K.J. (1985). Carbon Balance and Water Relations of Sorghum Exposed to Salt and Water Stress. Plant Physiol..

[29] Hwang Y., Morris J.T. (1994). Whole-plant Gas Exchange Responses of *Spartina alterniflora* (Poaceae) to a Range of Constant and Transient Salinities. Am. J. Bot..

[30] Moud A.M., Maghsoudi K. (2008). Salt Stress Effects on Respiration and Growth of Germinated Seeds of Different Wheat (*Triticum aestivum* L.) Cultivars. World J. Agric. Sci.

[31] Zou Y., Zhang Y., Testerink C. (2022). Root Dynamic Growth Strategies in Response to Salinity. Plant Cell Environ..

[32] Flowers T.J., Colmer T.D. (2008). Salinity Tolerance in Halophytes. New Phytol..

[33] Menezes-Benavente L., Kernodle S.P., Margis-Pinheiro M., Scandalios J.G. (2004). Salt-Induced Antioxidant Metabolism Defenses in Maize (*Zea mays* L.) Seedlings. Redox Rep..

[34] Kazemi K., Eskandari H. (2011). Effects of Salt Stress on Germination and Early Seedling Growth of Rice (*Oryza sativa*) Cultivars in Iran. Afr. J. Biotechnol..

[35] Shimizu K., Matsuoka Y. (2019). Redox Rebalance against Genetic Perturbations and Modulation of Central Carbon Metabolism by the Oxidative Stress Regulation. Biotechnol. Adv..

[36] Shahzad B., Fahad S., Tanveer M., Saud S., Khan I.A. (2019). Plant Responses and Tolerance to Salt Stress. Approaches for Enhancing Abiotic Stress Tolerance in Plants.

[37] Munns R., Tester M. (2008). Mechanisms of Salinity Tolerance. Annu. Rev. Plant Biol..

[38] Shabala S., Wu H., Bose J. (2015). Salt Stress Sensing and Early Signalling Events in Plant Roots: Current Knowledge and Hypothesis. Plant Sci..

[39] Tang X., Zhang H., Shabala S., Li H., Yang X., Zhang H. (2021). Tissue Tolerance Mechanisms Conferring Salinity Tolerance in a Halophytic Perennial Species Nitraria Sibirica Pall. Tree Physiol..

[40] Ozfidan-Konakci C., Yildiztugay E., Alp F.N., Kucukoduk M., Turkan I. (2020). Naringenin Induces Tolerance to Salt/Osmotic Stress through the Regulation of Nitrogen Metabolism, Cellular Redox and ROS Scavenging Capacity in Bean Plants. Plant Physiol. Biochem..

[41] Dai L., Li P., Li Q., Leng Y., Zeng D., Qian Q. (2022). Integrated Multi-Omics Perspective to Strengthen the Understanding of Salt Tolerance in Rice. Int. J. Mol. Sci..

[42] Jin T., An J., Xu H., Chen J., Pan L., Zhao R., Wang N., Gai J., Li Y. (2022). A Soybean Sodium/Hydrogen Exchanger GmNHX6 Confers Plant Alkaline Salt Tolerance by Regulating Na^+^/K^+^ Homeostasis. Front. Plant Sci..

[43] Wang C.-F., Han G.-L., Qiao Z.-Q., Li Y.-X., Yang Z.-R., Wang B.-S. (2022). Root Na^+^ Content Negatively Correlated to Salt Tolerance Determines the Salt Tolerance of *Brassica napus* L. Inbred Seedlings. Plants.

[44] Xiao L., Shi Y., Wang R., Feng Y., Wang L., Zhang H., Shi X., Jing G., Deng P., Song T. (2022). The Transcription Factor OsMYBc and an E3 Ligase Regulate Expression of a K^+^ Transporter during Salt Stress. Plant Physiol..

[45] Li X., Ye G., Shen Z., Li J., Hao D., Kong W., Wang H., Zhang L., Chen J., Guo H. (2023). Na^+^ and K^+^ Homeostasis in Different Organs of Contrasting Zoysia Japonica Accessions under Salt Stress. Environ. Exp. Bot..

[46] Ghassemi-Golezani K., Farhangi-Abriz S. (2021). Biochar-Based Metal Oxide Nanocomposites of Magnesium and Manganese Improved Root Development and Productivity of Safflower (*Carthamus tinctorius* L.) under Salt Stress. Rhizosphere.

[47] Gong W., Wang B.C. (2011). The Role of Ca^2+^ in Plant Response to Abiotic Stress. Chem. Life.

[48] Mustroph A., Albrecht G. (2003). Tolerance of Crop Plants to Oxygen Deficiency Stress: Fermentative Activity and Photosynthetic Capacity of Entire Seedlings under Hypoxia and Anoxia. Physiol. Plant..

[49] Cramer G.R., Ergül A., Grimplet J., Tillett R.L., Tattersall E.A.R., Bohlman M.C., Vincent D., Sonderegger J., Evans J., Osborne C. (2007). Water and Salinity Stress in Grapevines: Early and Late Changes in Transcript and Metabolite Profiles. Funct. Integr. Genom..

[50] Lambers H., Oliveira R.S., Lambers H., Oliveira R.S. (2019). Photosynthesis, Respiration, and Long-Distance Transport: Photosynthesis. Plant Physiological Ecology.

[51] Jacoby R.P., Millar A.H., Taylor N.L. (2013). Investigating the Role of Respiration in Plant Salinity Tolerance by Analyzing Mitochondrial Proteomes from Wheat and a Salinity-Tolerant Amphiploid (*Wheat* × *Lophopyrum elongatum*). J. Proteome Res..

[52] Li S., Li Y., Gao Y., He X., Zhang D., Liu B., Li Q. (2020). Effects of CO_2_ Enrichment on Non-Structural Carbohydrate Metabolism in Leaves of Cucumber Seedlings under Salt Stress. Sci. Hortic..

[53] Huang J., Zhang H., Wang J., Yang J. (2003). Molecular Cloning and Characterization of Rice 6-Phosphogluconate Dehydrogenase Gene That Is up-Regulated by Salt Stress A. Mol. Biol. Rep..

[54] Yuxi Z., Dan Y., Chunying L., Shupeng G. (2018). Dynamic of Carbohydrate Metabolism and the Related Genes Highlights PPP Pathway Activation during Chilling Induced Bud Dormancy Release in Tree Peony (*Paeonia suffruticosa*). Sci. Hortic..

[55] Guo S.R., Nada K., Katoh H., Tachibana S. (1999). Differences between Tomato (*Lycopersicon esuculentum* Mill.) and Cucumber (*Cucumis sativus* L.) in Ethanol, Lactate and Malate Metabolisms and Cell Sap PH of Roots under Hypoxia. J. Jpn. Soc. Hortic. Sci..

[56] Biemelt S., Keetman U., Albrecht G. (1998). Re-Aeration Following Hypoxia or Anoxia Leads to Activation of the Antioxidative Defense System in Roots of Wheat Seedlings. Plant Physiol..

[57] Muench D.G., Archibold O.W., Good A.G. (1993). Hypoxic Metabolism in Wild Rice (*Zizania palustris*): Enzyme Induction and Metabolite Production. Physiol. Plant..

[58] Kidrič M., Kos J., Sabotič J. (2014). Proteases and Their Endogenous Inhibitors in the Plant Response to Abiotic Stress. Bot. Serbica.

[59] Zadražnik T., Hollung K., Egge-Jacobsen W., Meglič V., Šuštar-Vozlič J. (2013). Differential Proteomic Analysis of Drought Stress Response in Leaves of Common Bean (*Phaseolus vulgaris* L.). J. Proteom..

[60] Wang Z.Q., Xu X.Y., Gong Q.Q., Xie C., Fan W., Yang J.L., Lin Q.S., Zheng S.J. (2014). Root Proteome of Rice Studied by ITRAQ Provides Integrated Insight into Aluminum Stress Tolerance Mechanisms in Plants. J. Proteom..

[61] Raessler M., Wissuwa B., Breul A., Unger W., Grimm T. (2010). Chromatographic Analysis of Major Non-Structural Carbohydrates in Several Wood Species–an Analytical Approach for Higher Accuracy of Data. Anal. Methods.

[62] Cate R.L., Roche T.E., Davis L.C. (1980). Rapid Intersite Transfer of Acetyl Groups and Movement of Pyruvate Dehydrogenase Component in the Kidney Pyruvate Dehydrogenase Complex. J. Biol. Chem..

[63] Abbasi G.H., Akhtar J., Anwar-ul-Haq M., Ali S., Chen Z., Malik W. (2014). Exogenous Potassium Differentially Mitigates Salt Stress in Tolerant and Sensitive Maize Hybrids. Pak. J. Bot..

[64] Büyük İ., Soydam-Aydin S., Aras S. (2012). Bitkilerin Stres Koşullarına Verdiği Moleküler Cevaplar. Turk. Bull. Hyg. Exp. Biol. Hij. Deney. Biyol..

[65] Kang G.Z., Li G.Z., Liu G.Q., Xu W., Peng X.Q., Wang C.Y., Zhu Y.J., Guo T.C. (2013). Exogenous Salicylic Acid Enhances Wheat Drought Tolerance by Influence on the Expression of Genes Related to Ascorbate-Glutathione Cycle. Biol. Plant..

[66] Koh J., Chen G., Yoo M.-J., Zhu N., Dufresne D., Erickson J.E., Shao H., Chen S. (2015). Comparative Proteomic Analysis of Brassica Napus in Response to Drought Stress. J. Proteome Res..

[67] Mishra K.K., Vikram P., Yadaw R.B., Swamy B.P.M., Dixit S., Cruz M.T.S., Maturan P., Marker S., Kumar A. (2013). QDTY 12. 1: A Locus with a Consistent Effect on Grain Yield under Drought in Rice. BMC Genet..

[68] Xu Z., Chen X., Lu X., Zhao B., Yang Y., Liu J. (2021). Integrative Analysis of Transcriptome and Metabolome Reveal Mechanism of Tolerance to Salt Stress in Oat (*Avena sativa* L.). Plant Physiol. Biochem..

