# Supplementary material for: Label-Free Proteomics Reveals the Response of Oat (Avena sativa L.) Seedling Root Respiratory Metabolism to Salt Stress
Source: Int J Mol Sci. 2025 Mar 14;26(6):2630. doi: 10.3390/ijms26062630 (PMC11942509; doi:10.3390/ijms26062630)
Supplement: Supplementary file 1 [file ijms-26-02630-s001.zip › ijms-3485287-supplementary.pdf]

Table S1. Differentially expressed proteins in the roots of two oat varieties after salt stress.

NO.	T3 vs T1		T5 vs T1		T4 vs T2		T6 vs T2		Up/down	Description
	log_2FC	*P* val.	log_2FC	*P* val.	log_2FC	*P* val.	log_2FC	*P* val.		
**Ion transport**										
**R7WFE8**	-3.804	0.002	-2.142	0.001	-1.414	0.010	-1.973	0.013	↓	Pollen-specific protein SF3
A0A3B6EBG0	1.628	0.018	1.100	0.037	2.594	0.002	2.925	0.000	↑	Uncharacterized protein
A0A287SEX0	1.302	0.011	1.219	0.019	1.735	0.006	1.840	0.005	↑	Uncharacterized protein
**Protein synthesis**										
A0A2X0SIM8	-1.544	0.004	-1.753	0.006	-1.435	0.002	-2.055	0.004	↓	Cytochrome b-c1 complex subunit Rieske, mitochondrial
A0A2S3I991	-2.517	0.002	-2.573	0.002	-2.555	0.003	-3.074	0.004	↓	Uncharacterized protein
A0A3B6DHS5	-1.901	0.022	-2.108	0.021	-1.038	0.031	-1.244	0.029	↓	Uncharacterized protein
I1IBS9	-1.308	0.000	-1.163	0.000	-1.720	0.022	-2.095	0.011	↓	Uncharacterized protein
A0A3Q7HQL5	2.260	0.000	1.816	0.031	1.587	0.042	1.952	0.002	↑	Uncharacterized protein
I1HYD6	-2.484	0.006	-2.737	0.005	-1.559	0.030	-1.885	0.018	↓	Signal recognition particle subunit SRP68
A0A453AMG5	3.955	0.042	3.513	0.016	3.458	0.020	4.322	0.002	↑	Uncharacterized protein
P59758	2.654	0.010	2.548	0.016	2.153	0.001	2.537	0.002	↑	30S ribosomal protein S13, chloroplastic
A0A287QKF3	3.216	0.001	3.273	0.030	4.218	0.003	4.066	0.016	↑	Uncharacterized protein
M8CAM5	-3.991	0.003	-4.176	0.006	-2.004	0.020	-4.139	0.012	↓	Heterogeneous nuclear ribonucleoprotein 27C
A0A2R6W6D4	2.635	0.014	2.006	0.040	1.466	0.008	1.429	0.017	↑	Uncharacterized protein
A0A1J7HK67	-1.224	0.007	-1.572	0.004	-1.072	0.033	-1.764	0.023	↓	Importin subunit alpha
I1I5M2	1.999	0.028	1.429	0.016	2.311	0.009	2.076	0.042	↑	Uncharacterized protein
A0A287HQI0	1.584	0.048	1.548	0.034	1.634	0.018	2.298	0.001	↑	TOG domain-containing protein
A0A3B6IKY0	-1.640	0.008	-1.303	0.014	-1.229	0.008	-1.007	0.007	↓	Uncharacterized protein
K3ZIY5	-5.403	0.025	-4.305	0.024	-2.919	0.038	-4.376	0.033	↓	Uncharacterized protein
**Antioxidant enzyme**										
J3MP40	-1.878	0.017	-2.871	0.017	-1.794	0.030	-2.819	0.035	↓	Peroxidase
M7ZH16	-1.891	0.010	-1.942	0.006	-1.897	0.003	-1.941	0.001	↓	Peroxidase
A0A2S3ISS8	1.973	0.003	1.653	0.026	2.661	0.021	3.246	0.021	↑	Glutamine synthetase
I1IFI7	-1.222	0.012	-2.032	0.006	-1.532	0.029	-2.494	0.025	↓	Glutamine synthetase
A0A2R6WCB6	3.838	0.004	3.312	0.000	3.225	0.011	3.882	0.007	↑	Succinate--CoA ligase [ADP-forming] subunit alpha, mitochondrial

A0A0Q3F350	1.100	0.035	1.172	0.002	2.164	0.007	2.572	0.011	↑	Catalase
A0A3B6PHD6	1.241	0.045	1.574	0.012	2.198	0.009	2.725	0.030	↑	Catalase
**Carbohydrates and Energy Metabolism**										
A0A287S5Q4	-1.303	0.017	-1.280	0.008	-1.288	0.014	-1.100	0.008	↓	Uncharacterized protein
A0A3Q7GF14	2.522	0.006	2.891	0.012	2.604	0.002	3.003	0.006	↑	6-phosphogluconate dehydrogenase, decarboxylating
A0A0Q3FHC7	-1.291	0.024	-1.107	0.029	-2.145	0.006	-2.729	0.003	↓	Nitrate reductase
A0A3B6N0D0	-2.741	0.000	-1.746	0.003	-1.443	0.017	-1.801	0.017	↓	Ferredoxin--NADP reductase, chloroplastic
M1B4S9	-2.915	0.003	-1.586	0.005	-1.561	0.031	-2.792	0.014	↓	Tubulin beta chain
D8RNM7	-2.369	0.018	-1.688	0.023	-2.549	0.032	-3.679	0.029	↓	Tubulin alpha chain
A0A0D2ZZ17	3.704	0.001	3.873	0.024	2.474	0.012	2.555	0.001	↑	Tr-type G domain-containing protein
R7QL58	2.114	0.000	1.882	0.005	2.072	0.012	2.181	0.010	↑	Elongation factor G, mitochondrial
I1HQW3	-2.775	0.016	-2.332	0.023	-2.111	0.001	-2.809	0.000	↓	Glycine cleavage system P protein
A0A0D3BYD7	-1.481	0.012	-1.312	0.006	-1.099	0.050	-2.130	0.025	↓	Fructose-bisphosphate aldolase
I1GZT2	-1.645	0.022	-1.351	0.030	-1.619	0.010	-2.735	0.011	↓	Oxidored_FMN domain-containing protein
T1NPH5	1.816	0.047	1.477	0.050	1.529	0.001	1.850	0.008	↑	Uncharacterized protein
A0A3B6FZW8	-1.150	0.000	-1.042	0.000	-1.006	0.048	-1.120	0.043	↓	ATP synthase subunit alpha
A0A161X8G8	-2.030	0.017	-2.298	0.013	-1.242	0.001	-4.065	0.000	↓	Uncharacterized protein
A0A2S3IEG9	-1.499	0.004	-1.474	0.002	-1.061	0.020	-2.377	0.008	↓	Alpha-mannosidase
W1NLP2	1.642	0.000	1.452	0.002	1.034	0.001	1.223	0.001	↑	2-C-methyl-D-erythritol 2,4-cyclodiphosphate synthase
M1AX28	-1.725	0.019	-2.835	0.004	-1.499	0.010	-3.083	0.012	↓	Uncharacterized protein
A0A287M227	2.824	0.011	2.595	0.043	2.632	0.008	2.576	0.038	↑	Delta-1-pyrroline-5-carboxylate synthase
A0A2K1WZ82	-1.198	0.002	-1.512	0.020	-1.537	0.007	-1.205	0.013	↓	Aldedh domain-containing protein
A0A287P1U7	-1.570	0.018	-1.847	0.016	-1.581	0.015	-1.632	0.009	↓	Alpha-L-AF_C domain-containing protein
A0A0Q3HC52	-1.743	0.001	-2.315	0.000	-1.195	0.002	-1.521	0.000	↓	GSDH domain-containing protein
A0A3B6SEK9	-2.308	0.000	-2.153	0.000	-1.303	0.007	-1.732	0.001	↓	GSDH domain-containing protein
A0A0E0LU23	2.118	0.004	1.641	0.036	1.355	0.038	1.813	0.022	↑	Glucose-1-phosphate adenylyltransferase
**Transcriptional regulation and signal transduction**										
Q85FR6	1.534	0.005	1.687	0.001	1.250	0.007	1.636	0.003	↑	DNA-directed RNA polymerase subunit beta-beta
K3XYQ8	-1.200	0.023	-1.009	0.025	-1.249	0.010	-1.145	0.014	↓	Uncharacterized protein
A0A287QVM8	1.306	0.016	1.814	0.018	1.413	0.018	1.178	0.023	↑	Transmembrane 9 superfamily member
A0A3B6B438	-1.343	0.014	-1.679	0.010	-1.455	0.003	-1.543	0.010	↓	Vacuolar ATPase assembly integral membrane protein VMA21 homolog

M0YD38	-1.904	0.000	-1.867	0.001	-1.390	0.001	-1.649	0.000	↓	Uncharacterized protein
A0A2K2CIK0	-1.477	0.014	-1.757	0.003	-1.193	0.040	-1.743	0.023	↓	Uncharacterized protein
**Secondary metabolism**										
M7ZHL0	2.898	0.000	2.698	0.049	2.576	0.003	2.875	0.000	↑	3-ketoacyl-CoA synthase 11
A0A453KE31	-2.314	0.002	-2.339	0.002	-1.140	0.047	-3.166	0.000	↓	Uncharacterized protein
**Unknown function**										
A0A0D9WD12	1.922	0.022	1.590	0.016	1.592	0.010	1.682	0.001	↑	Uncharacterized protein
A0A3B6CDW4	-1.026	0.006	-1.266	0.007	-1.314	0.003	-1.942	0.000	↓	Uncharacterized protein
M8BM31	-1.410	0.002	-1.168	0.009	-1.031	0.023	-1.579	0.010	↓	Uncharacterized protein
R7W9A6	-2.738	0.005	-1.573	0.003	-2.067	0.023	-2.287	0.022	↓	Uncharacterized protein
O22655	-2.249	0.003	-1.321	0.017	-1.220	0.033	-2.120	0.015	↓	Profilin-4
S6AWC2	-1.217	0.010	-1.328	0.007	-1.251	0.039	-1.262	0.033	↓	Cold induced 16

Table S2. Differentially expressed proteins in Bai2 root system after salt stress.

NO.	T3 vs. T1		T5 vs. T1		Up/ down	Description
	log_2FC	*P* val.	log_2FC	*P* val.		
**Ribosome**						
A0A453S7I4	1.069	0.027	1.006	1.069	↑	Elongation factor Ts, mitochondrial
A0A2T7EYR7	1.110	0.022	1.137	1.110	↑	40S ribosomal protein S6
M1BUI8	1.066	0.009	1.272	1.066	↑	40S ribosomal protein S8
A0A1D6KHX5	1.130	0.002	1.193	1.130	↑	Serine/arginine-rich splicing factor SC35
G7JLV1	-1.981	0.040	-2.353	-1.981	↓	Elongation factor 1-alpha
A0A0E0FY67	1.862	0.009	2.430	1.862	↑	Ribosomal_L7Ae domain-containing protein
A0A1R3I863	1.252	0.004	1.502	0.032	↑	60S ribosomal protein L36
A0A1D6HWQ4	2.387	0.004	2.688	0.011	↑	Uncharacterized protein
**Transcription**						
A0A0E0GLV8	-1.439	0.020	-1.438	0.023	↓	Protein kinase domain-containing protein
A0A1X7YHF8	1.127	0.013	1.005	0.027	↑	Multifunctional fusion protein
A0A0E0M3W3	-1.066	0.008	-1.163	0.006	↓	Uncharacterized protein
A0A251QL25	-2.874	0.007	-3.455	0.007	↓	Histone domain-containing protein
A0A453LHK0	1.133	0.029	1.109	0.021	↑	Alba domain-containing protein
A0A453S1Y0	1.401	0.004	1.331	0.001	↑	Uncharacterized protein
J3MRA4	-1.248	0.028	-1.811	0.019	↓	ATP-dependent DNA helicase
J3MMY0	-1.386	0.019	-1.361	0.009	↓	EF1_GNE domain-containing protein
**Signal transduction**						
A0A287JRY6	-2.202	0.011	-2.253	0.019	↓	Protein kinase domain-containing protein
A0A0D9WZ95	-1.473	0.001	-1.325	0.036	↓	PPM-type phosphatase domain-containing protein
A0A3B6GR47	-1.538	0.036	-1.483	0.012	↓	Usp domain-containing protein
A0A1D6FHD1	-1.029	0.023	-1.002	0.028	↓	Ubiquitin carrier protein 7
A0A1D6LVG0	-2.924	0.007	-2.782	0.004	↓	Blue copper protein
A0A287UPF3	-1.462	0.019	-1.414	0.026	↓	Phytocyanin domain-containing protein
**Secondary metabolism**						

M1ARA4	-1.707	0.001	-1.123	0.014	↓	Uncharacterized protein
A0A2K2CJP5	-1.089	0.033	-2.254	0.007	↓	Uncharacterized protein
A0A078HTM8	-2.148	0.022	-3.120	0.002	↓	BnaC03g33900D protein
**Post-translation modifications, protein turnover, molecular chaperones**						
A0A0Q3HRP0	-2.121	0.032	-1.742	0.026	↓	Proteasome subunit beta
A0A3B6KPR4	-1.607	0.002	-1.244	0.010	↓	Uncharacterized protein
M7ZZC1	-3.250	0.015	-1.326	0.048	↓	Protein-L-isoaspartate O-methyltransferase
A0A0E0JTM1	-1.139	0.022	-1.428	0.017	↓	Uncharacterized protein
J3LTH3	1.108	0.005	1.036	0.005	↑	Uncharacterized protein
A0A0E0KTJ9	-1.140	0.010	-1.786	0.007	↓	Uncharacterized protein
I1HEE4	-1.207	0.018	-1.397	0.004	↓	E2_bind domain-containing protein
M0RQY2	-2.540	0.005	-2.661	0.011	↓	HATPase_c domain-containing protein
A0A0D9VU88	-1.151	0.018	-1.175	0.020	↓	Peptide-methionine (R)-S-oxide reductase
M8BUH1	-1.112	0.013	-2.628	0.007	↓	Vacuolar-processing enzyme
A0A453JCW3	-2.945	0.022	-4.044	0.019	↓	Uncharacterized protein
I1HWJ6	-1.468	0.023	-1.520	0.003	↓	Peptidyl-prolyl cis-trans isomerase
A0A3B6PJ21	-2.611	0.017	-2.620	0.007	↓	SHSP domain-containing protein
A0A452Z9T3	-1.130	0.004	-1.173	0.003	↓	Uncharacterized protein
A0A453IDL2	-1.657	0.021	-1.275	0.029	↓	Uncharacterized protein
A0A2T7CCK3	1.157	0.030	1.108	0.004	↑	Mitochondrial fission 1 protein
K3Y9H3	-1.225	0.002	-1.257	0.005	↓	14_3_3 domain-containing protein
A0A2S3I1M0	-1.001	0.002	-1.042	0.004	↓	Uncharacterized protein
**Inorganic ion transport and metabolism**						
A0A1S3TKD5	-1.036	0.002	-1.086	0.003	↓	L-ascorbate peroxidase, cytosolic
A0A2T7DAI6	1.430	0.005	1.207	0.006	↑	PEROXIDASE_4 domain-containing protein
A0A0D9WRP4	-2.048	0.038	-1.515	0.048	↓	Uncharacterized protein
J3LA39	-1.263	0.021	-1.006	0.015	↓	Calcium-transporting ATPase
A0A3B6NNY2	1.164	0.016	1.323	0.001	↑	Uncharacterized protein
**Carbohydrate and energy metabolism**						
I1IX66	-2.015	0.008	-1.806	0.003	↓	Cytochrome b-c1 complex subunit Rieske,

						mitochondrial
D7M6L0	1.352	0.033	1.251	0.040	↑	Isocitrate dehydrogenase [NADP]
A0A3B5XTH2	1.350	0.045	1.340	0.023	↑	Oxidored_FMN domain-containing protein
F6HGH6	2.965	0.010	2.625	0.004	↑	FAD-binding PCMH-type domain-containing protein
I1GL66	-1.343	0.047	-1.497	0.031	↓	ETF domain-containing protein
M0T2D8	1.645	0.032	1.071	0.021	↑	Uncharacterized protein
A0A1D5WI33	-1.196	0.009	-1.054	0.004	↓	Uncharacterized protein
A0A0D9VPY1	5.427	0.006	5.285	0.010	↑	Uncharacterized protein
A0A287V4F1	-1.353	0.014	-1.121	0.030	↓	Germin-like protein
M7ZR32	-1.491	0.000	-1.506	0.000	↓	Germin-like protein
F2ELD1	-2.013	0.016	-2.159	0.009	↓	Fructose-bisphosphate aldolase
A0A2R6XU29	1.549	0.019	1.262	0.009	↑	PFK domain-containing protein
A0A287ST02	2.719	0.020	2.800	0.026	↑	MFS domain-containing protein
C5YQN5	-2.166	0.013	-2.953	0.019	↓	Alpha-L-AF_C domain-containing protein
A0A2S3I7B1	-1.878	0.003	-1.157	0.007	↓	Aldose 1-epimerase
I1H9A1	1.308	0.039	1.092	0.043	↑	Ribulose-phosphate 3-epimerase
I1PNA3	-1.061	0.013	-1.554	0.010	↓	Fn3_like domain-containing protein
I1GTQ7	-2.077	0.004	-2.117	0.007	↓	Uncharacterized protein
M8CEJ1	2.065	0.004	1.664	0.039	↑	Glucan endo-1,3-beta-glucosidase 3
**Components such as cytoskeleton and cell wall**						
A0A3B6EMQ5	-1.740	0.003	-1.798	0.002	↓	Uncharacterized protein
I1HTQ5	-1.435	0.001	-1.575	0.000	↓	Uncharacterized protein
K3ZV56	-1.446	0.003	-1.128	0.001	↓	Uncharacterized protein
F6GTE2	-2.399	0.020	-1.381	0.046	↓	Uncharacterized protein
A0A287HK37	1.377	0.012	1.441	0.009	↑	Sucrose synthase
V7BJB9	-1.912	0.010	-1.771	0.018	↓	Uncharacterized protein
K3XL85	-1.104	0.007	-1.504	0.001	↓	Uncharacterized protein
**Coenzyme transport and metabolism**						
A2XID1	-1.003	0.015	-1.098	0.001	↓	AB hydrolase-1 domain-containing protein
I1HHM3	-2.157	0.025	-1.134	0.043	↓	Uncharacterized protein

A0A251UB67	-1.474	0.002	-1.200	0.003	↓	S-adenosylmethionine synthase
A0A2T8KHT6	-1.544	0.024	-1.230	0.029	↓	S-adenosylmethionine synthase
M4FIV7	-2.761	0.013	-2.810	0.009	↓	S-adenosylmethionine synthase
Q307Y9	-2.768	0.000	-1.559	0.010	↓	S-adenosylmethionine synthase 1
A0A453CRV5	1.204	0.036	1.149	0.044	↑	Uncharacterized protein
A0A453F7Q7	3.658	0.007	3.587	0.029	↑	Uncharacterized protein
A0A3B5Y1X0	-1.037	0.003	-1.790	0.004	↓	BOWMAN_BIRK domain-containing protein
A0A0E0PEB1	-1.064	0.009	-1.074	0.004	↓	Uncharacterized protein
**Amino acid transport and metabolism**						
M0SR82	-3.836	0.000	-2.839	0.001	↓	Uncharacterized protein
A0A0D9XEF0	-2.008	0.040	-1.531	0.042	↓	Aminotran_1_2 domain-containing protein
**Unknown function**						
A0A3Q7E895	-1.459	0.005	-2.098	0.009	↓	Protein kinase domain-containing protein
A0A453F907	-1.980	0.008	-1.445	0.017	↓	WD_REPEATS_REGION domain-containing protein
A0A2K2CH24	1.159	0.022	1.112	0.010	↑	Uncharacterized protein
A0A2S3HZ11	-1.493	0.019	-1.949	0.017	↓	Uncharacterized protein
A0A3B6UC51	1.672	0.005	1.698	0.000	↑	C2 domain-containing protein
A0A287RUK5	-2.682	0.019	-3.236	0.017	↓	Purple acid phosphatase
K7U3S1	-1.593	0.011	-1.457	0.004	↓	Sucrose cleavage protein-like protein
M0XUE4	-1.016	0.002	-1.525	0.001	↓	SCP domain-containing protein
W1NIQ9	-1.034	0.036	-1.876	0.010	↓	Uncharacterized protein
A0A3B6NRZ2	1.177	0.026	1.600	0.008	↑	HMG box domain-containing protein
A0A0D9WXZ9	-1.198	0.008	-1.566	0.037	↓	Uncharacterized protein
A0A3B6ECS7	-1.006	0.022	-1.071	0.009	↓	Uncharacterized protein
K3XLC2	-1.483	0.014	-1.539	0.013	↓	Uncharacterized protein
F2DR57	1.246	0.006	1.072	0.026	↑	Predicted protein
A0A251QWU8	-2.623	0.010	-2.427	0.003	↓	Uncharacterized protein
K3ZDH3	-3.618	0.007	-5.242	0.007	↓	Uncharacterized protein
A0A2T7FAH7	1.203	0.029	1.367	0.019	↑	Uncharacterized protein
I1GSK5	-2.561	0.032	-2.844	0.035	↓	Uncharacterized protein

			**Antioxidant enzyme**			
A0A077RUR2	-2.819	0.012	-1.653	0.011	↓	Peroxidase
A0A0J8BPV6	-2.775	0.000	-2.999	0.001	↓	Peroxidase
A0A2T7DXQ7	-1.461	0.009	-1.424	0.015	↓	Peroxidase
A0A3B6RDG1	-1.917	0.005	-1.904	0.011	↓	Peroxidase
I1HF19	1.232	0.013	1.103	0.023	↑	Peroxidase
I1GSF9	-2.003	0.007	-1.981	0.001	↓	Uncharacterized protein

Table S3. Root fluorescence quantitative PCR template and primers.

Name	Sequence (5′ to 3′)	Length (bp)
A0A3B6PHD6	F: AGGTAGGCACGTGAAGTTCG<br>R: GCTGCGATCGATCTTTGCTG	252
I1I9A3	F: GCGCGCTTGCATGGTTATTA<br>R: CAGGAGGAATACACCGGAGC	160
A0A2T7DAI6	F: TTGTCGTTCTCCTTGAGGGC<br>R: ATCGAGGACCTCAACTCCCA	275
A0A2S3GZF9	F: TGGATGAAACTCTGCCAGCA<br>R: GTCGAGCAAGGTCGTGAGTA	220
M8A623	F: AGCAGGCTGTTTGTTGGAGT<br>R: GCAGAAGATGAGGAGAGGCC	123
A0A3B6B850	F: GCTATATGCAAGGGGTGGCA<br>R: ACCGAATTAGCCACCTGCAA	175
A0A1J7HFP8	F: ATATTGCGGTGGGATCGACC<br>R: TTTTCGCTGGTGTAAGCCCT	234
A0A3B6DHS5	F: GTTTGCCGGCCCTTTTTCAT<br>R: ACGTTCTCCCCTCTGCTACT	221
K3XV32	F: CGTAGGGCAACTGGTGGATT<br>R: TCAAGAAGCTCCAGGCCAAG	251
M1AX28	F: TAGAAATGGAAGTCGCGGGC<br>R: ACTTTCCCCACCCAAACTCG	177
A0A1D6QPT3	F: ACAGGAATGAAGGAGCCAGAG<br>R: ACACCATTACATACTTCCTGACACT	110
A0A3B6FZW8	F: ATCGCCGATCTCTCGTGAAC<br>R: GATAAACACACCGCCAAGCC	280
A0A0D9XIB2	F: TGGTTTGCCAATTGGTGCTG<br>R: CATGAGCACCAACTACGGGT	209

actin	F: CCAATCGTGAGAAGATGACCC<br>R: CACCATCACCAGAATCCAACA	135
